# AI-Driven Hyperspectral Imaging in Clinical Medicine: A Critical Appraisal of Translational Readiness

**DOI:** 10.3390/diagnostics16203338

**Published:** 2026-10-14

**Authors:** Asma Begam Mohamed Meeran, Muhammad E. H. Chowdhury, Mansura Naznine, Sawal Hamid Md Ali, Rajendran Sankaran, Shona Pedersen

**Affiliations:** 1 Department of Electrical Engineering, Qatar University, Doha 2713, Qatar; am2300474@qu.edu.qa (A.B.M.M.); p159922@siswa.ukm.edu.my (M.N.); sawal@ukm.edu.my (S.H.M.A.); 2 Department of Electrical, Electronic and Systems Engineering, Faculty of Engineering and Built Environment, Universiti Kebangsaan Malaysia, Bangi 43600, Malaysia; 3 Environmental Science Center, Qatar University, Doha 2713, Qatar; srajendran@qu.edu.qa; 4 Department of Basic Medical Sciences, College of Medicine, QU Health, Qatar University, Doha 2713, Qatar; spedersen@qu.edu.qa

**Keywords:** hyperspectral imaging, deep learning, spectral-spatial models, surgical guidance, digital pathology, clinical translation

## Abstract

The instruments used for intraoperative guidance have not improved over the years. Traditional margin evaluations fail in 10–20% of cancer surgeries, and histopathology confirmation can take 48–72 h. A promising alternative is hyperspectral imaging (HSI), which allows for real-time, label-free, non-contact, and detailed biochemical characterization of tissues. This review synthesizes 130 studies from 2015 to 2026, identified through a systematic search across multiple databases following PRISMA-ScR guidelines. The studies cover various clinical areas, including surgical oncology (53%), neurosurgery (10.8%), perfusion assessment (6.2%), gastroenterology (5.4%), dermatology (4.6%), and emerging domains (20%). Each clinical area is evaluated using a four-part Translational Readiness Appraisal framework. This framework shifts the focus from technical proficiency to deployment evidence by examining hardware compatibility with operating rooms, real-time workflow integration, cross-site validation, and regulatory readiness. Since 2015, the combination of clinically approved HSI hardware and artificial intelligence has led to a tenfold increase in publications. However, a significant gap remains: none of the reviewed studies meet the evidence criteria necessary for regulatory approval. Of the studies, 48.5% show strong single-site validation, but 50.8% remain in the proof-of-concept stage. The heavy reliance on intra-patient validation tends to inflate performance estimates, leaving issues like calibration across devices and regulatory pathways largely unaddressed. HSI has demonstrated technical maturity in several areas, particularly in head and neck margin assessment and brain tumor delineation. The primary reason for the lack of clinical adoption is not imaging performance but rather the absence of multicenter validation and a formal regulatory evidence framework.

## 1. Introduction

Precision medicine rests on a deceptively simple proposition: that the right information, available at the right moment, changes outcomes. Yet for all the sophistication of modern oncology, the surgical theater remains a conspicuous exception. Missed positive margins occur in 10–20% of oncological resections [1], directly affecting reoperation rates, adjuvant treatment burden, and long-term disease-free survival [2]. Standard histopathology requires 48–72 h, far too slow for intraoperative guidance [3]. Frozen section analysis takes 20 to 40 min out of the surgical workflow and needs cryostat equipment that is not available in many surgical suites [4]. Only tumors with approved fluorescent probes can be treated with fluorescence-guided surgery, which is essentially target-dependent [5]. Although Raman spectroscopy provides large amounts of spectral data, it does not have the speed and spatial coverage necessary for comprehensive intraoperative tissue evaluation. None of these methods provides surgeons with what they really need: a real-time, biochemically detailed view of tissue without any labels at the time of decision [6].

HSI addresses this need by capturing continuous spectral signatures across hundreds of narrow bands [7], enabling non-contact and label-free assessment of tissue physiology without exogenous contrast agents or tissue destruction [8]. As an optical reflectance-based modality, HSI differs fundamentally from cross-sectional imaging techniques such as computed tomography (CT), magnetic resonance imaging (MRI), and ultrasound, which rely on X-ray attenuation, magnetic resonance, and acoustic reflection, respectively. HSI is therefore suited to surfaces and tissues accessible to optical illumination, including intraoperative surgical fields, endoscopic surfaces, dermatological tissue, retinal imaging, and ex vivo histopathological specimens.

Advances in HSI have expanded its application from remote sensing to biomedical imaging, with early studies focusing on identifying pathological spectral differences and tissue-specific signatures [9]. However, the high dimensionality of HSI data presents challenges for conventional analysis and direct clinical interpretation. Machine learning (ML) and deep learning (DL) have addressed these challenges by enabling automated feature extraction and pattern recognition [5]. In particular, convolutional neural network (CNN)-based methods have been applied to disease biomarker detection [10], tumor margin assessment [11], tissue classification [12], and intraoperative guidance [13]. This progression has shifted biomedical HSI from an imaging modality primarily used for tissue characterization toward a data-driven approach with potential to support clinical decision-making.

Recent studies have reported promising results with AI-enabled HSI for tissue assessment [14], non-invasive detection [15], and automated optical biopsy [16]. Despite these advances, high model performance does not necessarily indicate clinical readiness. On the other hand, recent prospective studies have reported weaker cross-center generalization [17], limited patient diversity [18], and challenges in translating findings from animal models to humans [19]. Hybrid deep learning and self-supervised learning frameworks have further reduced dependence on large labeled datasets, which is an important advantage in a field where annotated clinical data remain scarce. However, despite these methodological advances, prospective clinical evidence remains limited [20].

Independent validation and integration into clinical practice remain important barriers to translation [21]. The brain tumor benchmark highlighted the importance of patient-level evaluation [22], while the continued use of intra-patient validation can produce optimistic performance estimates by limiting exposure to inter-patient spectral variability [23]. Device-dependent calibration, batch effects, and site-specific spectral variation may further affect generalizability [24]. Beyond model validation, clinical adoption requires integration with existing workflows [25], prospective evaluation [26], and regulatory assessment [27]. HSI-derived tissue oxygenation markers, for example, still require interventional clinical trials to establish their value for hemodynamic management [28]. Regulatory adoption is another underdeveloped area, with only isolated studies reporting CE certification or discussing specific regulatory considerations and no identified studies providing formal evidence of regulatory submission [20]. Thus, the literature continues to show a gap between technical model performance and real-world translational readiness, particularly in independent validation, workflow integration, and regulatory evaluation.

Together, these gaps contribute to selection and publication bias and indicate the need for future studies that address clinical validation, device and site variability, workflow integration, and regulatory requirements from the study-design stage. To address these gaps, this review synthesizes clinical HSI literature from 2015 to 2026, guided by five central research questions:

1. What specific clinical needs are currently targeted by HSI, and what are the operational limitations of established tools for intraoperative margin assessment, tissue characterization, and real-time diagnosis?
2. What is the landscape of publicly available and proprietary biomedical HSI datasets (2015–2026) regarding tissue diversity, annotation standards, spatial-spectral resolution, cohort sizes, acquisition conditions, and research accessibility?
3. What are the primary methodological and clinical evidence gaps in published HSI literature, particularly concerning cohort sizes, validation protocols (inter- vs. intra-patient), histopathological ground truth matching, device dependency, and publication bias?
4. How do current medical HSI applications perform when systematically evaluated across a four-domain appraisal framework comprising Operating Room hardware compatibility, real-time workflow integration, cross-site validation, and regulatory/health economic feasibility?
5. What prioritized technical, clinical, and regulatory steps are required to transition HSI from an experimental technology into routine surgical deployment?

**Figure 1:**
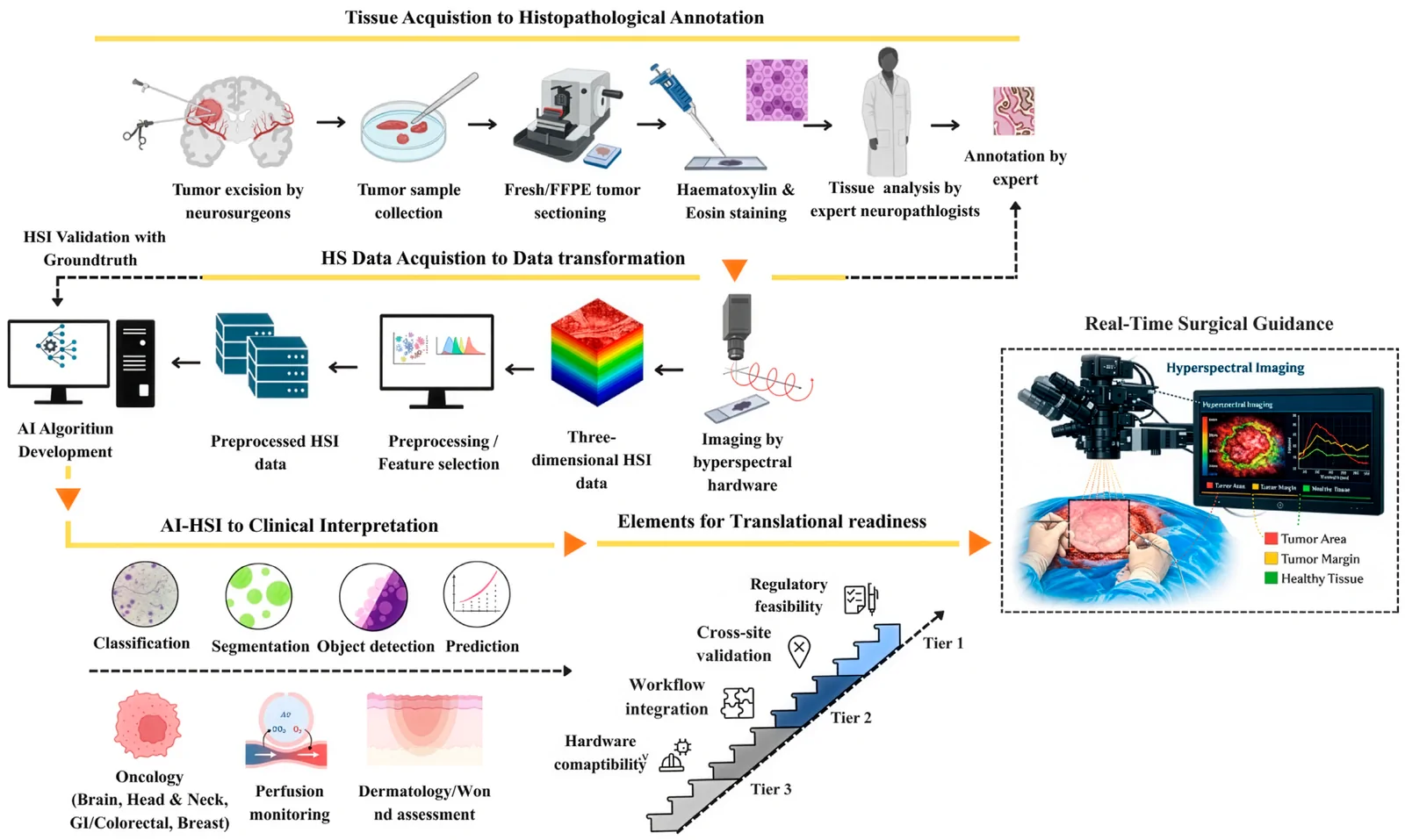
End-to-end clinical workflow and key translation barriers for AI-guided medical hyperspectral imaging.

By addressing these five research questions, this review provides a systematic, evidence-based synthesis that bridges optical AI innovations with real-world surgical utility. To visually conceptualize this end-to-end translation, Figure 1 presents a comprehensive schematic mapping the full clinical, technical, and regulatory journey of medical hyperspectral imaging. The overarching headings above each yellow highlighted line mark the sequential progression of tasks required to move from initial tissue harvesting to real-time intraoperative execution. Because the workflow unites several distinct methodologies and clinical steps into a single framework, its core components and operational flow are detailed below to aid interpretation:

- Tissue Acquisition to Histopathological Annotation: The upper pipeline illustrates the standard pathological ground truth establishment. Beginning with neurosurgical tumor excision, fresh or formalin-fixed paraffin-embedded (FFPE) tissue sections are prepared and stained with haematoxylin and eosin (H&E). Expert neuropathologists subsequently analyze and annotate these slides, defining the reference diagnostic boundaries required to train and validate optical models.
- HS Data Acquisition to Data Transformation: Running parallel to the pathological pathway, the technical imaging pipeline begins with hardware-based hyperspectral data collection, capturing rich three-dimensional HSI cubes. These cubes undergo spectral preprocessing and feature selection before feed-forwarding into specialized AI algorithms, where model outputs are rigorously cross-validated against the expert neuropathological ground truth.
- AI-HSI to Clinical Interpretation: The lower-left block details the primary machine learning tasks—ranging from tissue classification and spatial segmentation to object detection and predictive analytics. These computational outputs directly support diverse clinical domains, including oncological margin assessment (across brain, head and neck, GI/colorectal, and breast tumors), tissue perfusion monitoring, and wound care.
- Real-Time Surgical Guidance and Translational Readiness: As depicted on the right, the clinical culmination of this pipeline is real-time intraoperative guidance, where augmented HSI overlays clearly delineate the tumor core, tumor margins, and healthy surrounding tissue directly within the surgeon’s field of view. The accompanying staircase model maps the translational progression required for widespread adoption, prioritizing hardware compatibility (Tier 3) and seamless workflow integration (Tier 2) before advancing to cross-site validation and regulatory clearance (Tier 1).

The subsequent section details the unique aspects of the methodology contributed by each of the research questions.

## 2. Methodology of the Review

This review was conducted as a critical structured narrative review in accordance with the PRISMA-ScR (Preferred Reporting Items for Systematic Reviews and Meta-Analyses extension for scoping reviews) guidelines. This framework was selected because the 130 included studies spanned heterogeneous acquisition hardware, analytical methods, clinical areas, and outcome metrics that made statistical pooling impossible. In these cases, structured narrative synthesis is the best method to use. Study selection and data extraction were performed using the predefined criteria described below, with a standardized extraction form applied consistently across all the included studies.

### 2.1. Search Strategy and Databases

A systematic literature search was performed across several electronic databases, such as PubMed, Scopus, IEEE Xplore, ScienceDirect, Springer Link, Wiley, and other sources covering January 2015 to February 2026. This window captures the pre- and post-deep learning eras of medical HSI within a single analytical framework. The lower limit is the start of clinical HSI studies driven by CNNs, and the upper limit includes the newest certified hardware systems and transformer-based architectures. The primary Boolean search string was (“hyperspectral imaging” OR “spectral imaging” OR “medical hyperspectral imaging”) AND (“medical imaging” OR “biomedical optics” OR “clinical application”) AND (“diagnosis” OR “surgical guidance” OR “pathology” OR “cancer”) AND (“tissue classification” OR “deep learning” OR “machine learning”). A secondary refinement search incorporating terms such as “band selection,” “spectral unmixing,” “hyperspectral image analysis,” “segmentation,” and “delineation” was applied to capture domain-specific analytical literature. Conference proceedings from SPIE Photonics West were hand-searched for eligible full-text contributions.

### 2.2. Article Selection Criteria

All eligibility criteria were pre-specified and applied sequentially: first at the title and abstract level, then by full-text review. Table 1 details the full inclusion and exclusion criteria followed in this review.

Following the study selection process, the included literature was examined to characterize the overall development and publication landscape of HSI in clinical medicine. The next section summarizes the distribution and progression of the identified studies across publication years, journals, publishers, clinical applications, and other key characteristics.

### 2.3. Study Selection and Prisma Flow

**Figure 2:**
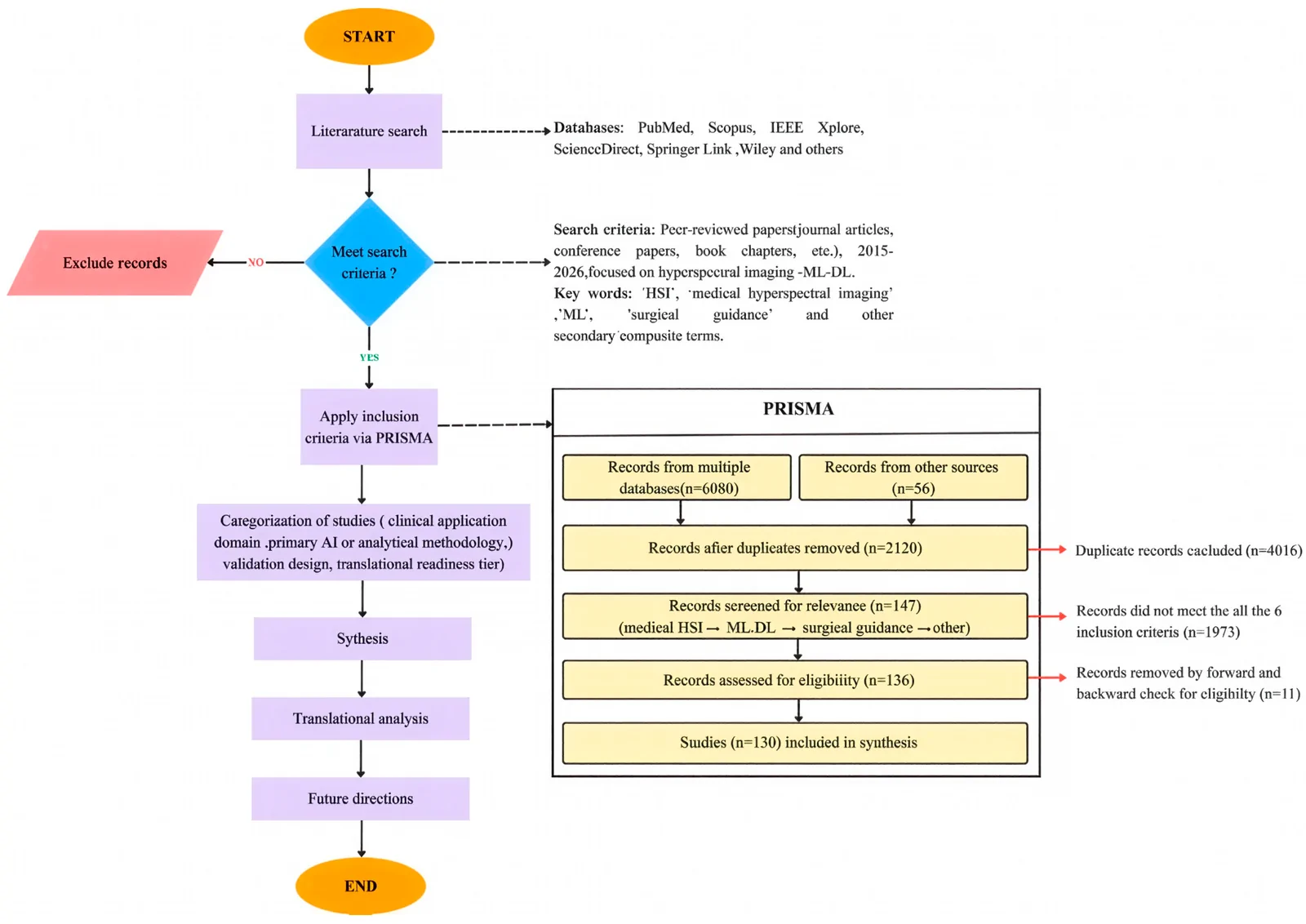
PRISMA-guided workflow illustrating database search, screening, eligibility assessment, and final study inclusion.

Deduplicating the records that were retrieved from the aforementioned databases reduced the 6136 original records to 2120 unique citations prior to screening. Following a relevance screening process based on the six inclusion criteria, 147 records were selected for full-text review. Of these, 136 were evaluated for eligibility; after forward and backward citation checks, 6 were eliminated, leaving 130 studies that satisfied all inclusion criteria and were moved on to synthesis. Figure 2 depicts the entire selection process using the PRISMA-ScR flow diagram. Among the 130 included studies were original research articles (*n* = 114, 87.7%), followed by conference papers (*n* = 10, 7.7%), systematic reviews (*n* = 5, 3.8%), and one book chapter (*n* = 1, 0.8%).

**Figure 3:**
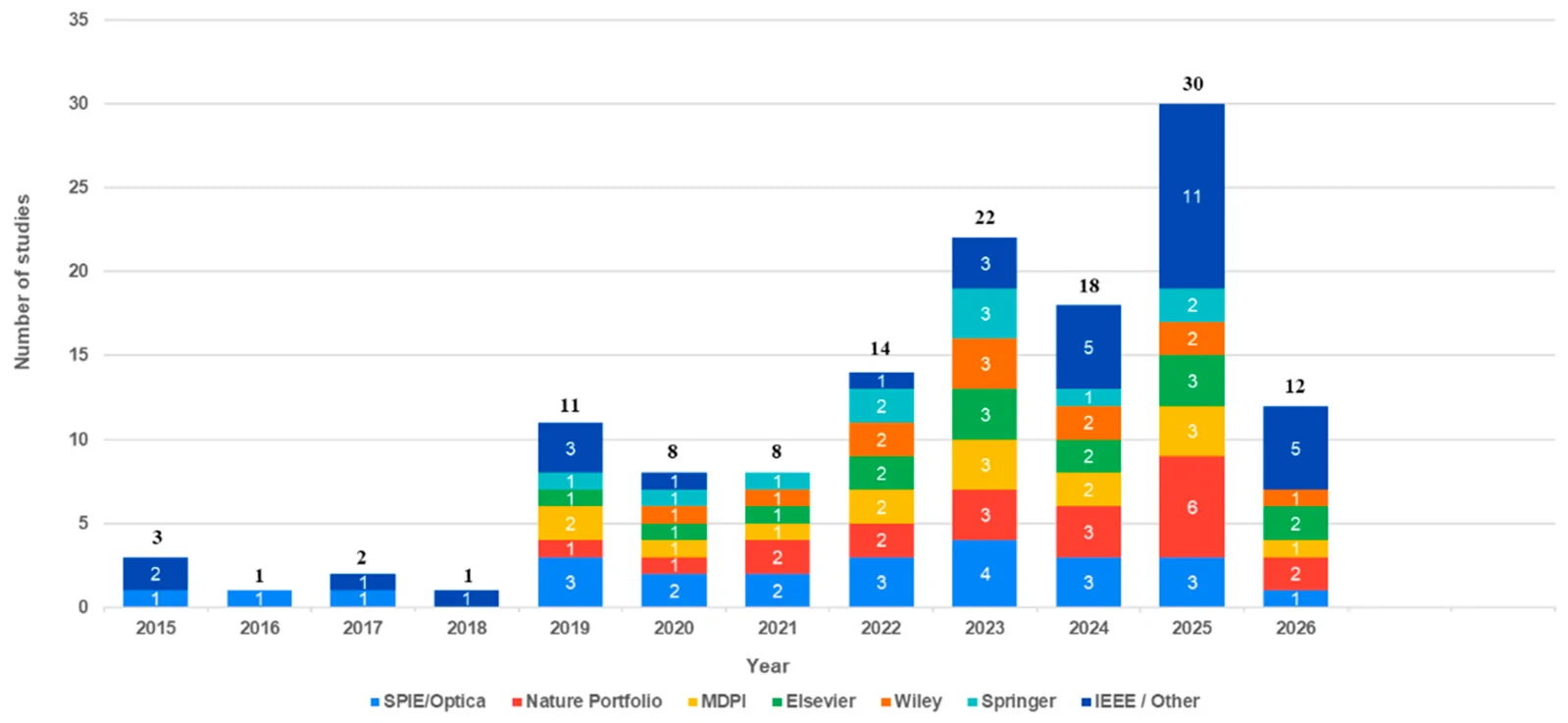
Illustration of temporal distribution of studies (2015–2026), showing a consistent rise in research activity and growing adoption of HSI in clinical applications.

To describe the growth trajectory of the field, a bibliometric analysis was performed across the 130 included studies. Three dimensions were examined: annual publication volume, journal and publisher distribution, over time (Figure 3). Publication volume increased approximately tenfold between 2015 and 2025, from three papers in 2015 to a peak of 30 in 2025, with the steepest inflection occurring between 2019 and 2023. Data for 2026 represent a partial year (January–February only) and should not be interpreted as indicative of annual output.

**Figure 4:**
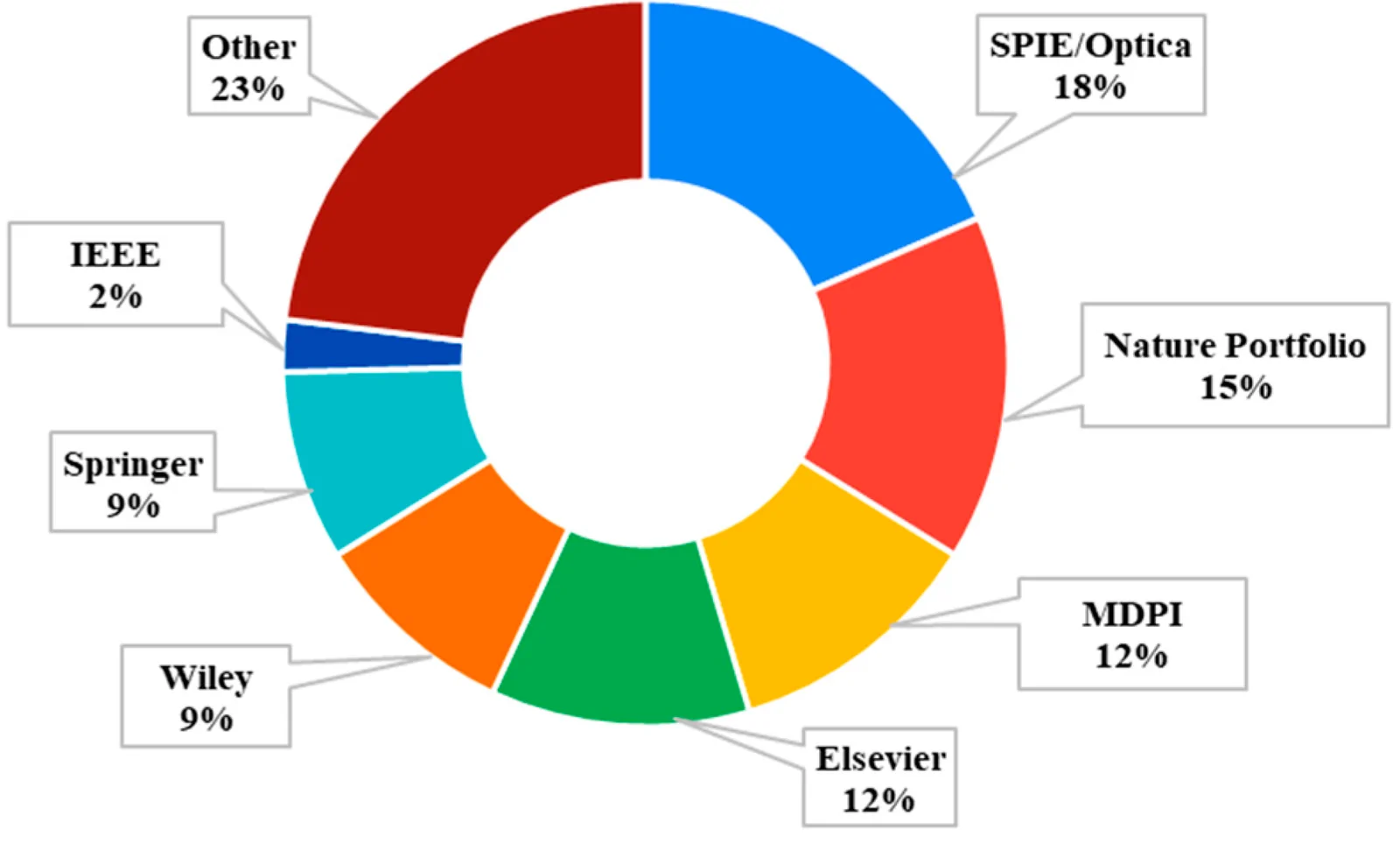
Publisher-wise distribution of selected articles.

Figure 4 illustrates the distribution of publications by publisher across the reviewed HSI-AI clinical literature. SPIE/Optica represented the largest single publisher group, accounting for 18% of the publications, reflecting the strong contribution of the biomedical optics community to clinical HSI research. Nature Portfolio accounted for 15% of the total publications and entered the reviewed literature after 2019. MDPI and Elsevier each contributed 12%, followed by Springer (9%), with the remaining publications distributed across Wiley, IEEE, and other publishers.

This distribution highlights the multidisciplinary nature of clinical HSI-AI research, spanning biomedical optics, medical imaging, engineering, and clinical research communities. The following sections describe how these studies were classified, how evidence quality was assessed, and how the Translational Readiness Appraisal (TRA) framework was structured.

### 2.4. Data Extraction and Classification

A standardized extraction form was applied to the 130 included studies. Studies were classified across five dimensions: clinical application, primary AI or analytical methodology, dataset characteristics, validation design, and translational readiness tier. The primary clinical focus areas included surgical oncology (*n* = 69), neurosurgery (*n* = 14), perfusion and tissue viability assessment (*n* = 8), gastroenterology (*n* = 7), dermatology (*n* = 6), and other specialized or multi-domain clinical areas (*n* = 26). Because some studies addressed overlapping clinical applications or evaluated tissues across different experimental settings, including preclinical models, in vivo clinical studies, and ex vivo tissue specimens, each study was assigned according to its primary medical target. Evidence quality was assessed separately using four levels: Level I (multicenter prospective study), Level II (single-center prospective study with inter-patient validation), Level III (retrospective study with inter-patient validation), and Level IV (intra-patient validation or proof-of-concept).

### 2.5. Translational Readiness Appraisal Framework

The central evaluative contribution of this review is the application of a four-domain Translational Readiness Appraisal framework to each clinical application area. The design developed to address the specific gap in prior HSI reviews has been extensively assessed for technical performance under experimental conditions, while its readiness for clinical deployment has not been evaluated. The four domains and their operational definitions are listed in Table 2. Each clinical application domain was assigned a Translational Readiness Tier on a three-point scale. Tier 1 indicates sufficient evidence across all four domains to support regulatory submission. Tier 2 reflects evidence in one or two domains, with multi-site validation absent. Tier 3 designates proof-of-concept status only. This tiering system forms the structured basis for the domain-level appraisals in Section 8.

**Figure 5:**
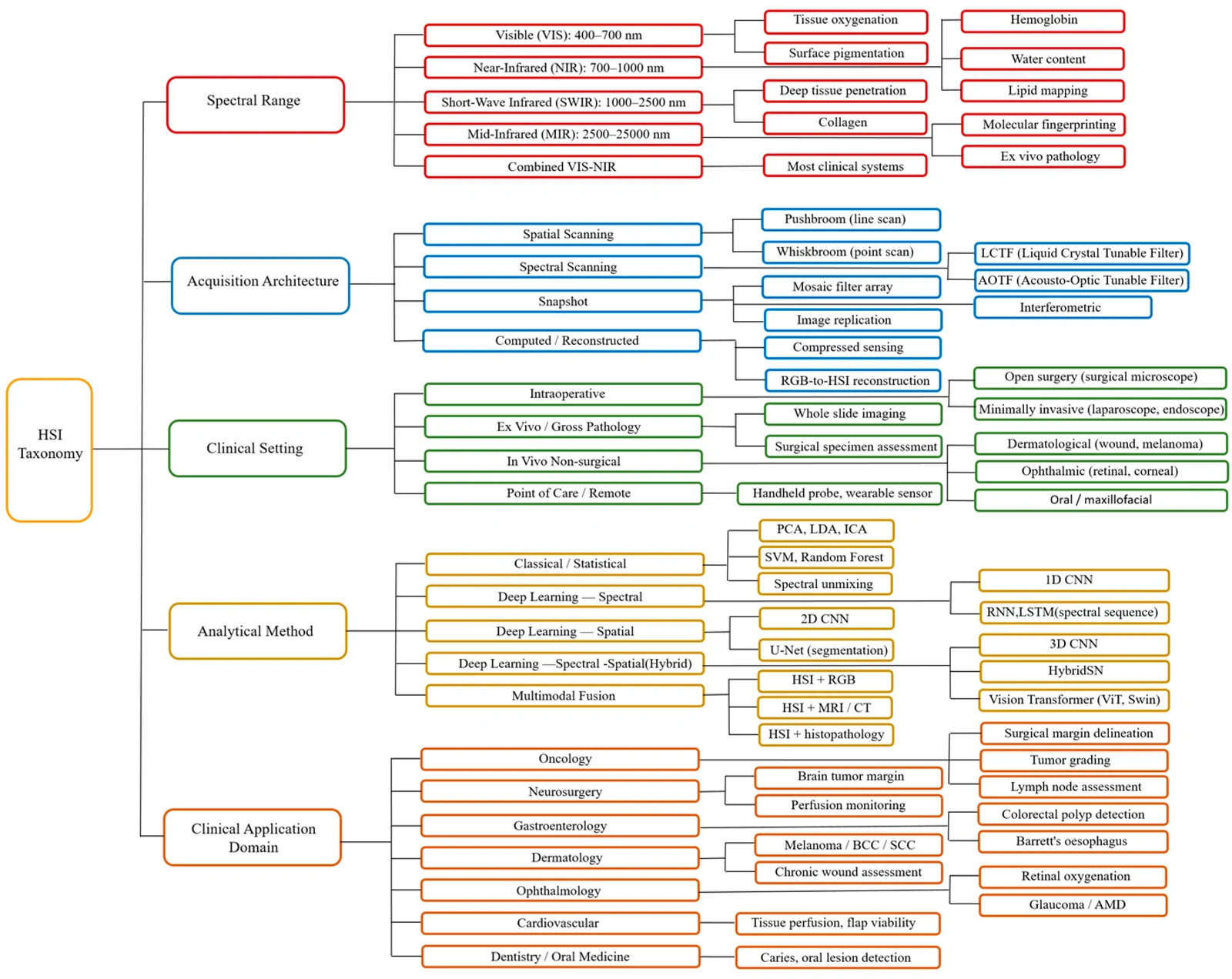
Comprehensive taxonomy of hyperspectral imaging in clinical medicine across five dimensions: spectral range, acquisition architecture, clinical setting, analytical method, and application domain.

Figure 5 depicts a detailed taxonomic map across the five dimensions examined in this review. Five interconnected elements define the scope of clinical HSI research. Spectral range—spanning visible, near-infrared, and short-wave infrared regions—determines tissue penetration depth and the biological information available for analysis. Acquisition architecture, encompassing pushbroom, snapshot, and filter-based systems, governs the trade-off between spectral resolution and acquisition speed, directly influencing real-time clinical viability. Clinical setting distinguishes four operational contexts—intraoperative, ex vivo, in vivo outpatient, and preclinical—each carrying distinct technical and regulatory requirements. Analytical model reflects the field’s evolution from classical statistical methods and handcrafted spectral features toward advanced deep learning architectures, a progression that determines generalization capacity and clinical interpretability. Finally, clinical application domain illustrates the breadth of HSI investigation across oncological tissue detection, perfusion monitoring, wound assessment, retinal imaging, and oral diagnostics, demonstrating that HSI is advancing across multiple clinical pathways simultaneously. Together, these five elements provide a structured entry point for understanding how HSI research is organized and where the critical gaps to clinical translation remain.

## 3. Imaging Methodology

Hyperspectral imaging produces a three-dimensional data cube (x, y, λ) by combining spatial imaging with spectral dispersion optics, such as prisms, gratings, or liquid crystal tunable filters [29]. When tissue is illuminated, the reflected, scattered, or emitted light is spectrally resolved across dozens to hundreds of narrow, contiguous wavelength bands [30]. Unlike standard RGB imaging, which integrates light into three broad and overlapping color channels, or multispectral imaging (MSI), which samples a limited number of discrete wavelength bands, HSI records a detailed spectral signature at each spatial pixel [31]. Table 3 summarizes the key technical differences among these modalities, including their respective acquisition outputs: a two-dimensional color image for RGB, discrete spectral bands for MSI, and a three-dimensional hyperspectral cube for HSI.

**Figure 6:**
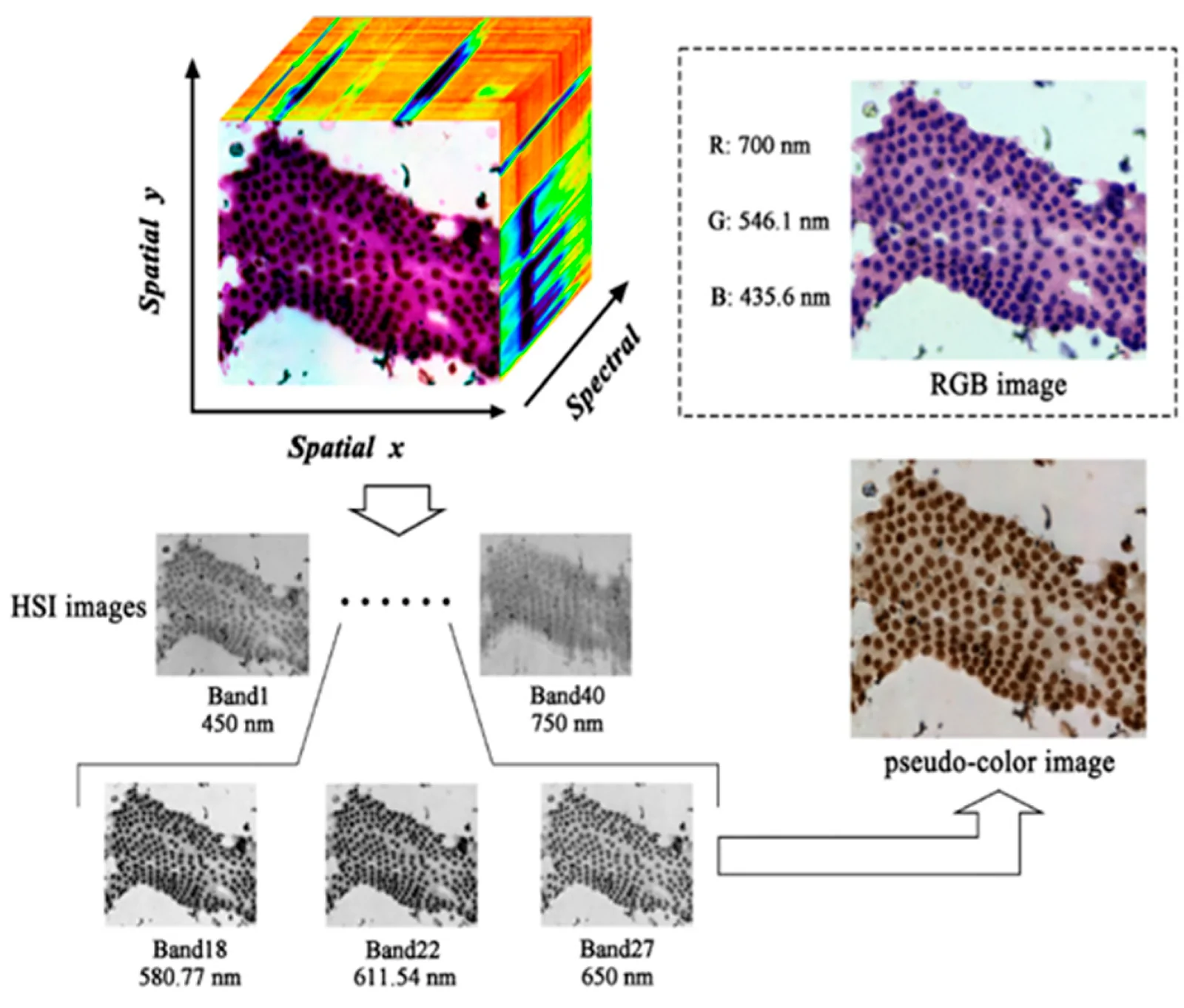
Comparison of a microscopic hyperspectral data cube and a corresponding H&E-stained RGB image. Adapted from Qin et al. [36].

The main clinical advantage of this high spectral resolution is its sensitivity to subtle molecular and physiological variations [32]. Key tissue chromophores, including oxyhemoglobin, deoxyhemoglobin, lipids, and water, exhibit distinct spectral absorption profiles that contribute to tissue-specific signatures [33,34]. These spectral differences can enable HSI to distinguish pathological, neoplastic, or ischemic tissue from surrounding healthy tissue, even when they appear visually similar under standard surgical lighting [35]. This is explained in Figure 6, which illustrates the hyperspectral data cube acquired using a microscopic HSI camera and the corresponding H&E-stained RGB image from the same field of view [36]. The HS data cube contains 40 spectral bands, including wavelengths that are not visible to the human eye, whereas the conventional RGB image contains only three visible channels: red, green, and blue. Since the full hyperspectral cube cannot be directly displayed as a conventional color image, three representative bands (Bands 18, 22, and 27) are selected to generate a pseudo-color HS image. The H&E-stained image is presented as an RGB image using its red, green, and blue channels. The figure therefore illustrates the greater spectral dimensionality of HSI compared with conventional RGB imaging.

**Figure 7:**
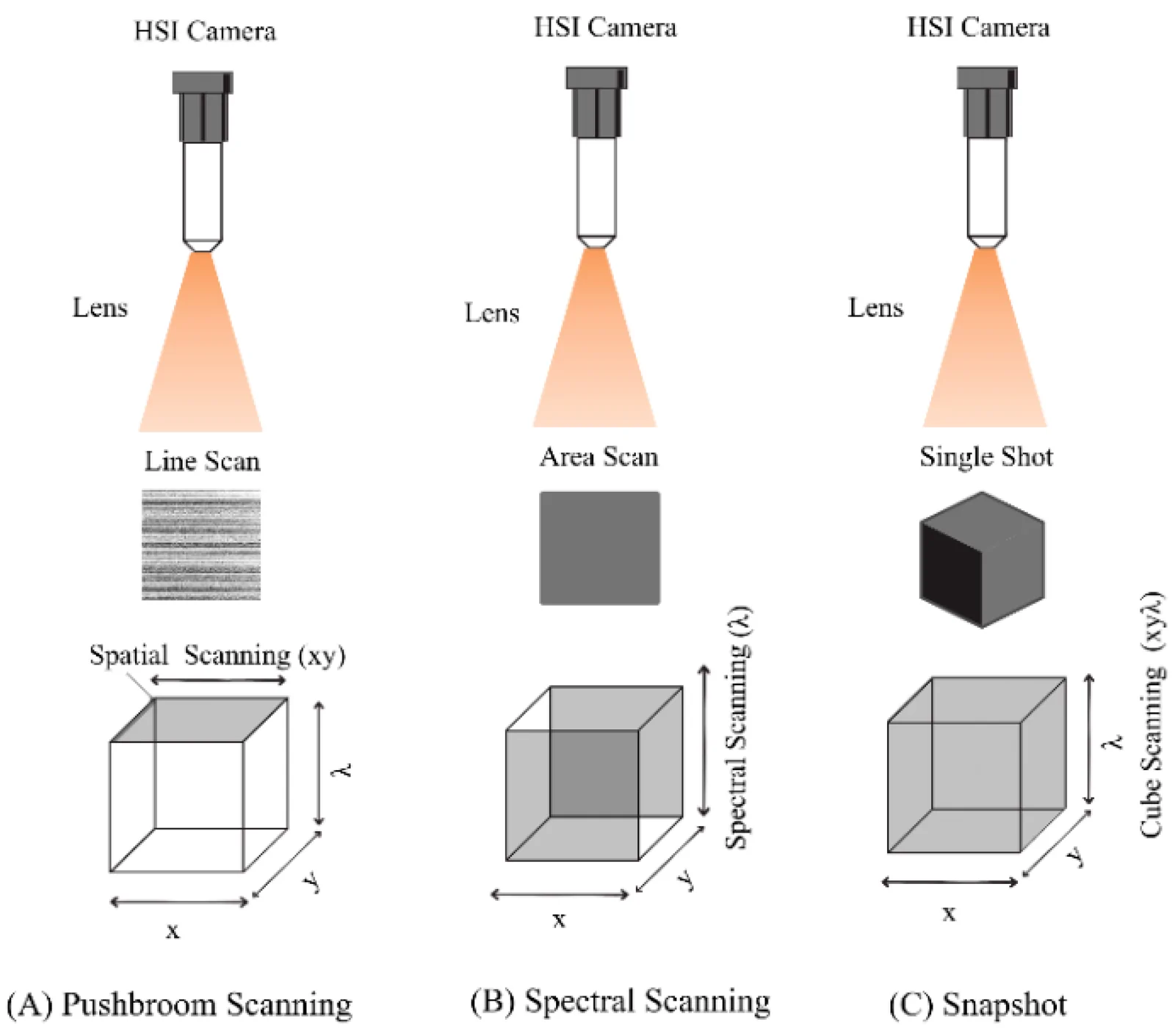
Principal acquisition architectures in medical hyperspectral imaging (x, y—spatial axis; λ—spectral axis).

Hyperspectral imaging systems typically acquire between visible to Mid-Infrared range (≈400–25,000 nm) and several hundreds of spectral bands, depending on the acquisition architecture and spectral range. Across the 130 reviewed studies, the visible–near-infrared (VIS–NIR) range (≈400–1000 nm) predominates and generally used with median of 128 spectral bands in clinical HSI. Because it encompasses important tissue absorption features while remaining compatible with compact optical detectors [29]. Within this spectral range, medical HSI systems primarily employ three major acquisition architectures (Figure 7), each offering distinct trade-offs in spatial and spectral resolution, acquisition speed, and system complexity that influence their clinical applicability [10]. These architectures are described in the following subsections.

### 3.1. Pushbroom Scanning

A pushbroom system scans tissue line by line, capturing full spectral information per line, whereas a motorized stage or sensor displacement builds the spatial image sequentially [19]. The spectral resolution is high (typically 2–5 nm); however, the sequential acquisition is sensitive to motion. This makes pushbrooms well-suited for stabilizing ex vivo specimens and controlled intraoperative fields but poorly suited for pulsating or moving tissues [28]. The most common pushbroom platform in this review was the TIVITA Tissue system [31], which was used in perfusion and intraoperative oncology studies [32,37]. Its clinical footprint is a reflection of the architecture’s advantages and disadvantages.

### 3.2. Snapshot

Snapshot systems are built with CMOS mosaic filter platforms that can capture the entire hypercube in one exposure, with acquisition times of less than 10 ms [33]. This speed makes snapshots the only architecture genuinely compatible with moving in vivo tissue, which explains its dominance in endoscopic, retinal, and intraoperative perfusion applications within this review [34]. The trade-off is the resolution. Since detector pixels are shared across both spatial and spectral dimensions, the resolution in either the spatial or spectral dimension, or both, is lower than in scanning systems. In recent years, this gap has been reduced but not completely closed by sensor engineering.

### 3.3. Wavelength-Scanning

Systems that use either liquid crystal tunable filters (LCTF) or acousto-optic tunable filters (AOTF) take one spectral frame at a time by stepping through different wavelengths in order [35]. The CRI Maestro, an LCTF-based system, appeared in several earlier studies in this review [23]. Acquisition times approaching one minute per hypercube render these systems impractical for real-time guidance. Their value lies in controlled ex vivo and histological settings, where time pressure is absent and spectral flexibility is a priority [7]. These trade-offs, summarized in Table 4, directly determine translational feasibility.

Acquisition strategies that require sequential scanning are not suitable for in vivo real-time clinical use. This implies that regardless of how well the algorithms function, they cannot be applied in real-world scenarios. The diversity in acquisition and scanning strategies directly impacts dataset characteristics, motivating a discussion on publicly available hyperspectral imaging datasets.

## 4. Publicly Available Datasets

The availability of publicly accessible hyperspectral imaging datasets in clinical and surgical research remains limited but is steadily expanding across multiple tissue domains and acquisition settings. Across the studies reviewed, spectral band counts range from as few as 25 to as many as 826, with a median of 128 and a mode of 100 bands reflecting the dominance of pushbroom camera systems in current acquisition pipelines. The majority of datasets (60.8%) were acquired ex vivo under controlled laboratory conditions, while only 37.7% involved in vivo acquisition, underscoring a translational gap between current dataset composition and real-world intraoperative demands. Ground truth labeling is predominantly histopathological (67.7%), representing the accepted gold standard for tissue classification, though a clinically notable 6.2% of studies provide no clear annotation source, a reproducibility concern that limits cross-study comparability.

Neuro-oncology was the first field to work on public datasets [22]. The HELICoiD project released the first publicly available in vivo brain tumor HSI dataset [37], which made it possible to separate glioblastomas and set the first standard for validating machine learning in surgery across patients [38]. In head and neck oncology, the HSPath-Bench dataset introduced the first public microscopic HSI resource for squamous cell carcinoma, supporting reproducible computational pathology and segmentation model development [39]. The HeiPorSPECtRaL dataset has addressed large-scale multi-organ coverage [19]. It contains thousands of annotated intraoperative porcine HSI frames from different organ classes and is currently one of the largest publicly available surgical HSI resources for organ recognition tasks [25].

A dataset of 174 patients with cholangiocarcinoma has helped with scene-level classification and spectral interval convolution research in gastrointestinal and hepatobiliary pathologies [40]. This is one of the largest disease-specific pathology HSI benchmarks to date. Breast oncology has provided the first publicly accessible dataset on breast cancer recurrence through TCIA, facilitating spectral biomarker studies for margin evaluation and recurrence forecasting [41]. Kopriva et al. released the most recent HSI dataset for liver metastatic colorectal cancer. It has pixel-level tumor annotations, a high spectral resolution of 1 nm, and pseudo-RGB co-registered image features [42]. With these datasets, the key features found in the reviewed dataset literature are summarized in Table 5, and all publicly available datasets are found in Table 6.

Despite this progress, dataset heterogeneity in spectral resolution, acquisition settings, annotation quality, and disease focus continues to challenge cross-study comparisons and model generalizability. Expanding publicly available datasets with consistent annotations, multi-organ coverage, and multi-institutional data collection is therefore essential for advancing clinically deployable hyperspectral imaging systems.

## 5. Data Transformation Techniques

Before evaluating the translational readiness of HSIs in specific clinical areas, it is essential to establish a shared understanding of the process by which raw spectral data are transformed into clinically actionable results. Following the establishment of the hardware environment and acquisition characteristics, the next step in downstream modeling focuses on data transformation techniques. It converts raw hyperspectral data into noise-reduced, model-ready, and analytically meaningful input.

### 5.1. Preprocessing

Raw hyperspectral cubes cannot be fed directly into a classifier. Before any analysis, a preprocessing pipeline must strip out the physical artifacts introduced by uneven lighting, sensor drift, and tissue preparation; the order and rigor of that pipeline matter enormously [48]. However, less than one-third of the 130 included studies provide sufficient detail on their white-reference acquisition to allow for reproduction. That omission is one of the main reasons models trained in one lab fail when moved to another. Despite varying implementations, the reviewed studies consistently follow four sequential stages.

Illumination correction: Compensates for non-uniform lighting across the tissue surface, a real problem in surgery where working distance, illumination angle, and instrument interference change moment to moment.

Radiometric calibration: Converts raw sensor counts into reflectance values using white and dark references. Without this, it is impossible to compare spectra from various sessions or devices, and batch effects become inevitable.

Noise reduction: Cleans the spatial and spectral domains via spectral smoothing, or spatial filtering. Spectral normalization is typically applied as a complementary preprocessing step to reduce intensity variations and standardize spectral distributions across pixels [49].

Dimensionality reduction: Reduces hyperspectral data cubes containing hundreds of spectral bands to a more compact feature space (≈5 to 30 spectral bands or components) before downstream analysis or deep learning. It is an important preprocessing step and has been implemented using a range of approaches across the reviewed studies.

**Figure 8:**
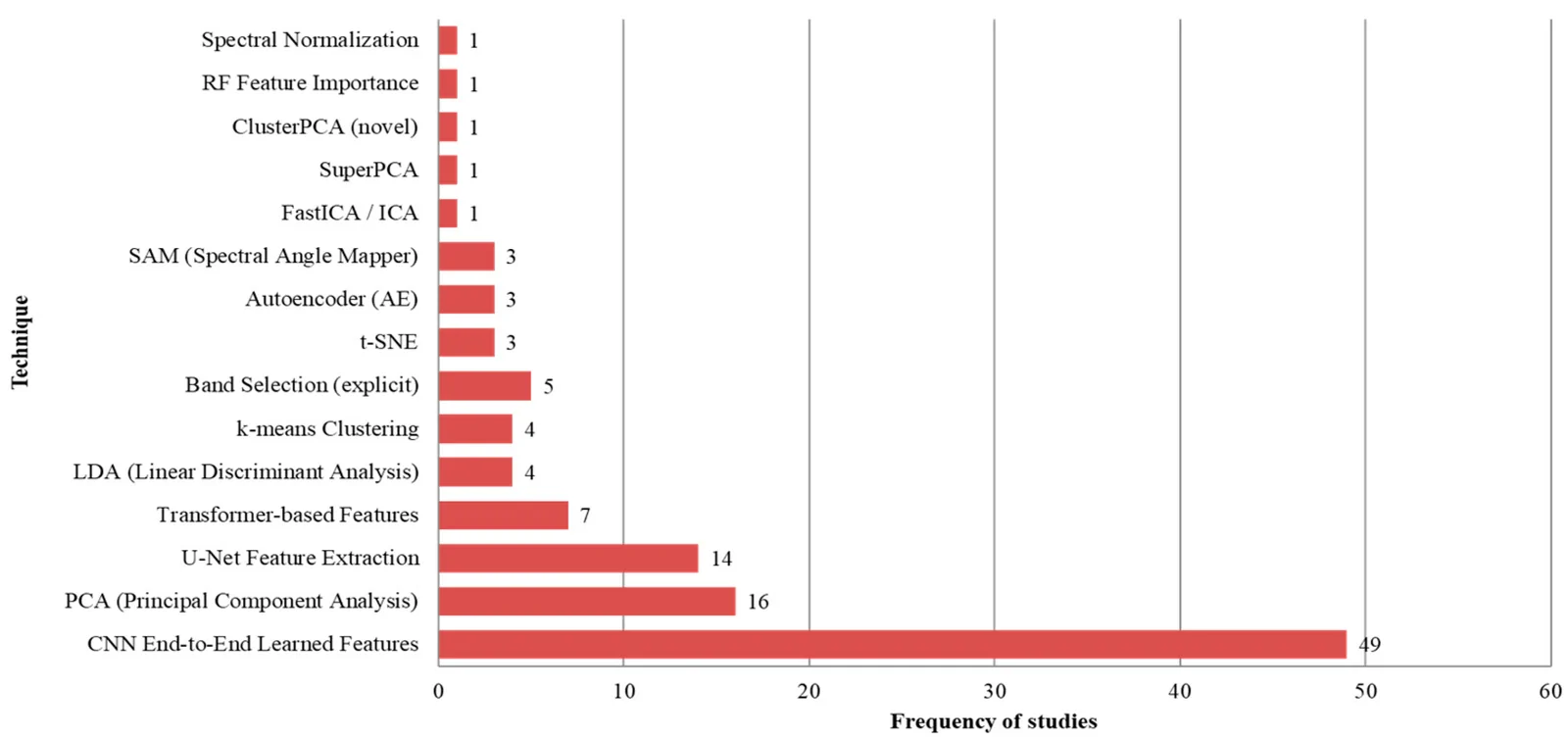
Preprocessing and feature extraction methods used in the studied literature.

Figure 8 summarizes the quantitative distribution of feature extraction and dimensionality reduction methods reported across the reviewed literature. Rather than relying solely on classical manual techniques, 49 studies employed CNN End-to-End Learned Features, allowing deep convolutional kernels to compress high-dimensional spatial-spectral cubes automatically within latent layers. Among traditional transformation techniques, Principal Component Analysis (PCA) predominated (16 studies), supplemented by novel variants such as SuperPCA (*n* = 1) and ClusterPCA (*n* = 1). U-Net Feature Extraction (*n* = 14) and Transformer-based Features (*n* = 7) served as popular deep learning compression strategies for segmentation and long-range spectral modeling, respectively. Furthermore, Explicit Band Selection was utilized in five studies to isolate a small subset of diagnostic wavelengths, while other studies adopted Linear Discriminant Analysis (LDA, *n* = 4), Autoencoders (AE, *n* = 3), t-SNE (*n* = 3), Spectral Angle Mapper (SAM, *n* = 3), FastICA (*n* = 1), and Random Forest Feature Importance (*n* = 1). Together, these strategies highlight a clear transition in medical HSI from manual linear band compression toward end-to-end spatial-spectral representation learning.

While these foundational preprocessing techniques standardize signal quality, transforming high-dimensional cubes into computationally efficient, diagnostic representations relies on two specialized paradigms: Band Selection and Spectral Unmixing. As highlighted in Table 7, ten of the reviewed articles specifically examine the evidence for band selection and primary preprocessing. Notably, Aloupogianni et al. and Tkachenko et al. are among the few studies that treat preprocessing choices as controlled experimental variables rather than fixed decisions [50,51]. Because band selection directly informs hardware miniaturization and real-time intraoperative execution, its specific algorithmic mechanisms and performance trade-offs are examined in detail in the following dedicated subsection.

### 5.2. Band Selection

**Figure 9:**
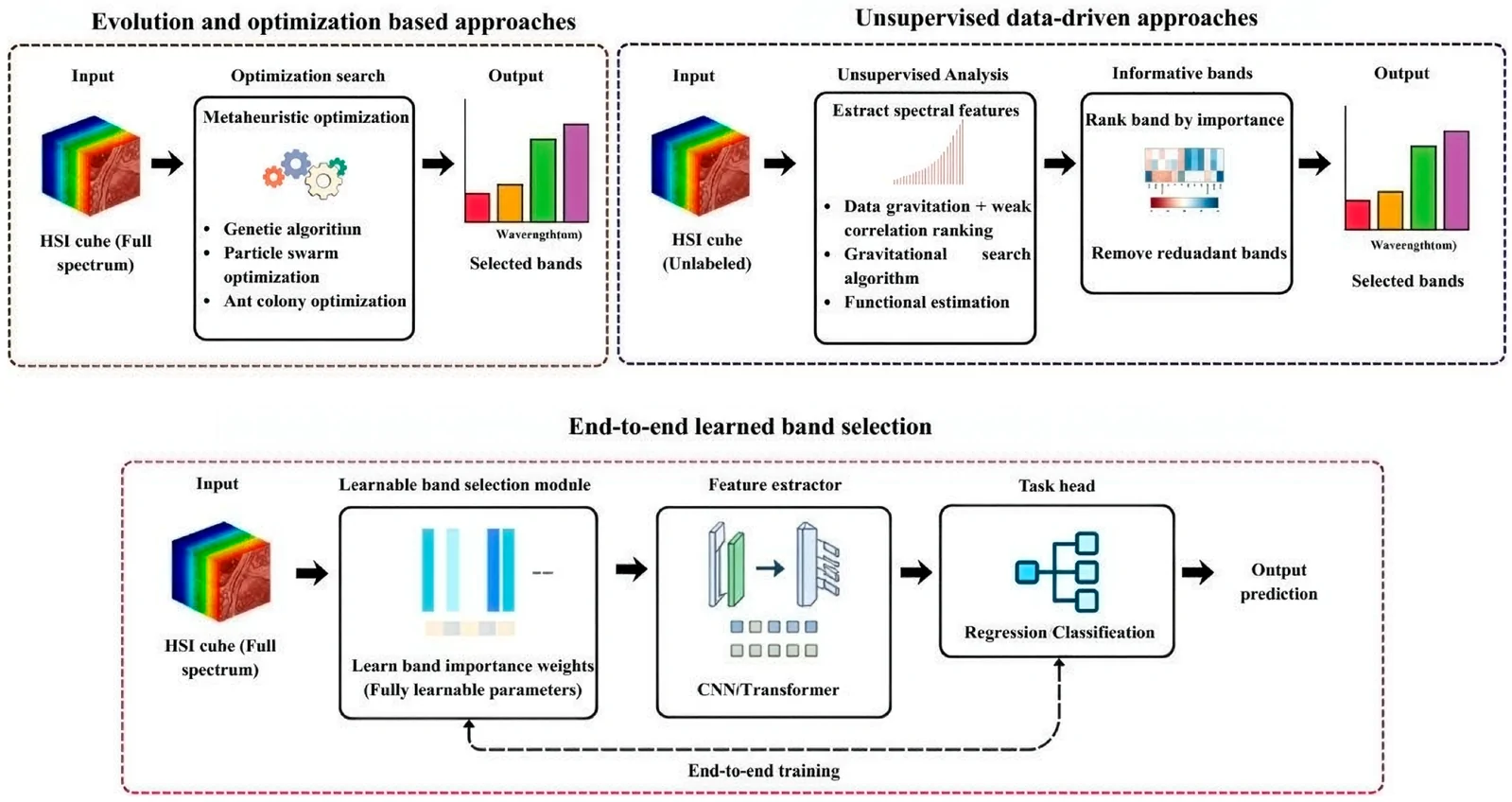
Conceptual workflows of band selection techniques in medical hyperspectral imaging.

Across the 130 reviewed studies, the number of spectral bands captured by medical hyperspectral imaging systems ranges widely from 25 to 826 bands, exhibiting high device-dependent variability. While the overall dataset median stands at 12 bands (driven by snapshot systems with fewer channels), the distribution peaks at a mode of 100 bands, reflecting the widespread clinical adoption of standard pushbroom line-scanning cameras (Table 5). Six studies treat band selection as their primary research question rather than a preprocessing detail. They fall into three categories that represent a clear methodological progression. Figure 9 shows these band selection strategies in HSI, illustrating the shift from separate, optimization-based selection toward integrated, learning-based frameworks. The workflows emphasize differences in supervision requirements, computational strategy, and integration with downstream models.

**Evolutionary and optimization-based approaches:** These approaches represent the earliest systematic band selection work in medical HSI. Martinez et al. utilized the HELICoiD intraoperative brain dataset to compare three metaheuristic optimization algorithms: the genetic algorithm, particle swarm optimization, and ant colony optimization [52]. In comparison to the full-spectrum baseline, all three improved tumor sensitivity by 20% while reducing the entire spectral set to about 48 bands. This result directly translates into faster acquisition and inference, both of which are necessary for intraoperative real-time use. However, the drawback is that evolutionary algorithms require labeled data to guide the optimization and are computationally expensive during the selection phase. When annotated clinical datasets are small, it is difficult to meet this constraint.

**Unsupervised data-driven approaches:** Zhang et al. addressed the labeling problem with DGWCR, a hybrid algorithm that combines data gravitation (measuring inter-sample attraction by spectral distance and density) with weak correlation ranking (eliminating redundant bands) [53]. Applied to the HELICoiD brain database, DGWCR achieved 99.17% overall accuracy with an SVM using no labeled training data. A follow-up GSA-UBS [54], a discretized gravitational search algorithm, exceeded 90% accuracy across three patients’ data. Both studies, however, were validated exclusively on HELICoiD, a limitation worth noting [55]. Ayala et al. used wrapper-based Best First Search with Random Forest regression to determine which wavelengths are specifically required for oxygenation quantification, taking a functional rather than a classificatory approach to band selection [56]. Their finding that 10 to 20 carefully chosen bands can reproduce the oxygenation estimates of a full 101-band spectrum with the same mean absolute error is very important for perfusion monitoring. When oxygenation is the most important clinical parameter, it provides a reason based on evidence for switching from hyperspectral to multispectral acquisition. This could lower hardware costs and acquisition times without significantly lowering diagnostic accuracy.

**End-to-end learned band selection:** This approach is the most recent and possibly the most clinically useful method because it includes selection in the classification architecture instead of treating it as a separate step. Shi et al. presented SpecNet applied to breast fibroepithelial lesion differentiation. SpecNet reached AUC 0.89, with spectral importance weights mapping onto known biochemical absorption features in the 540–590 nm and 700–820 nm ranges [57]. As part of the HSPath-Bench framework for head and neck histopathology, Zhang et al. introduced Maximum Mass-based Band Selection (M^2^BS), which selects bands by maximizing spectral mass distribution differences between tissue classes under sparse supervision. Combined with an ALGFF CNN-Swin Transformer classifier, M^2^BS achieved up to 89.96% overall accuracy with fewer than 50% of available training labels [39]. Chen et al. assessed the SAVE algorithm, which reconstructs narrow-band endoscopic images from optimized RGB-equivalent band combinations, specifically for esophageal cancer detection. They discovered that band-optimized SAVE reconstruction improved the accuracy of object detection to about 90.9% compared to regular white-light imaging [58].

Four of the six primary band selection studies use brain tumor datasets, driven by the public availability of HELICoiD rather than any clinical priority for neurosurgery. Only one study addresses wound perfusion monitoring and endoscopic HSI, the two domains where band reduction would have the most immediate translational impact. More critically, no study has demonstrated that a chosen band set generalizes across acquisition systems. Until that is proven, selected bands remain potentially device-specific signatures and not clinically portable spectral features. Band selection and preprocessing are interdependent decisions: the bands retained determine which calibration steps are necessary, yet the two are rarely optimized together.

**Table 7:** Primary preprocessing and band selection studies across the literature.

Clinical Domain	Dataset	Primary Contribution	Method/Algorithm	Task	Best Performance	Key Limitation/Gap	Reference
Brain/Neuro-oncology	In vivo brain data (HELIcoID)	Band selection	Gravitational Search Algorithm—unsupervised band selection (GSA-UBS) + SVM/KNN/LDA classifiers	Tissue classification	>90% OCA (Overall Classification Accuracy) across 3 brain datasets	Evaluation criteria for band quality are uncertain, having been tested on only 3 patients, and real-time implementation is unvalidated	[54]
Brain/Neuro-oncology	In vivo brain data (HELIcoID)	Band selection	Data Gravitation + Weak Correlation Ranking (DGWCR), unsupervised; no labeled data required	Tissue classification	99.17% OCA (SVM, dataset 13-01)	Optimal band count undetermined; 3-patient dataset; no clinical outcome validation	[53]
Brain/Neuro-oncology	In vivo brain data (HELIcoID)	Band selection	Genetic Algorithm, Particle Swarm Optimization, and Ant Colony Optimization compared for spectral band reduction	Tissue classification	~48 bands retained; tumor sensitivity +20% vs. full spectrum	Only 4 patients with labeled tumor pixels; no inference speed benchmarking reported	[52]
Intraoperative perfusion/functional	In vivo H&N tumor data (mouse model)	Band selection	Wrapper-based Best First Search + Random Forest regression; Monte Carlo simulation validation	Perfusion assessment	10–20 bands reproduce 101-band oxygenation MAE	Validated only in mouse models; no human clinical trial; spectral range limited to oxygenation-relevant bands	[56]
Gastroenterol/Endoscopy	In vivo esophageal cancer images (dysplasia vs. SCC)	Band selection	SAVE algorithm—RGB-to-NBI reconstruction via optimized band selection; YOLOv5/YOLOv8/SSD classifiers	Cancer detection	SSD precision ~90.9%; F1 ~86.05%	Dataset imbalance; no paired WLI/NBI from the same lesion; band optimality not generalized beyond esophageal cancer	[58]
Head and neck oncology	Head and neck squamous cell carcinoma specimens (HSPath-Bench)	Band selection	Maximum Mass-based Band Selection (M2BS) + ALGFF CNN-Swin Transformer hybrid: sparse supervision	Histopathological classification	OA up to 89.96%, Kappa > 0.84	ROI-based only; whole-slide imaging not implemented; single-center dataset	[39]
Dermatology/Skin oncology	Pigmented skin lesions data	Dimension reduction	11 methods compared: PCA, FastICA, RICA, LDA, SuperPCA, ClusterPCA, RFI, AE, etc., + SVM classifier	Classification and segmentation	Jaccard 47% (RFI/no reduction best); RFI and baseline outperformed dedicated methods	19 HSI cubes only; low sensitivity across all methods; no DL model tested; no color standardization	[50]
GI/Colorectal oncology	Colorectal cancer specimens	Preprocessing	Systematic evaluation: standardization vs. normalization, patch size, sample/class weighting, blood filtering: 3D-CNN backbone	Pixel-wise classification	AUC up to 0.90; MSS up to 0.778 with optimized preprocessing	56 patients ex vivo only; smoothing/filtering degraded performance; no in vivo validation	[51]
Head and neck oncology		Super-resolution	RGB-guided unsupervised CNN (modified U-Net) for SR reconstruction + Inception-CNN for classification	Image reconstruction and classification	AUC up to 0.94; reconstructed HR-HSI outperforms original HSI and RGB	Small dataset; relies on simulated low-resolution HSI for training; no in vivo validation	[59]
Brain/Neuro-oncology		Calibration	Hardware calibration, white reference protocol, automated scanning, RGB-HSI co-registration; no AI classification applied	Standardizing HSI/histopathological capturing procedure	High spatial correlation coefficients; methodology validated on tissue samples	No clinical diagnostic accuracy reported; large-scale validation not demonstrated; single-site feasibility only	[26]

Figure 9 illustrates the three approaches to band selection in hyperspectral imaging. The top-left block shows evolution and optimization-based approaches, where metaheuristic algorithms such as genetic algorithm, particle swarm optimization, and ant colony optimization search through the full spectral cube to identify the most informative wavelength bands. The top-right block shows unsupervised data-driven approaches, which extract spectral features from unlabeled HSI data using methods such as data gravitation and gravitational search algorithms, then rank bands by importance and remove redundant ones to produce a reduced band selection. The bottom block shows end-to-end learned band selection, where a fully learnable band selection module is trained jointly with a CNN or transformer-based feature extractor and a regression or classification task head, allowing the model to learn which bands are most useful for the target task directly from data without a separate selection step. Together, these three paradigms represent the progression from manual search-based selection to fully automated learning-based approaches in HSI dimensionality reduction.

### 5.3. Spectral Unmixing

Spectral unmixing operates as a third analytical pathway, positioned between raw data and clinical output and separate from both preprocessing and classification. Instead of labeling each pixel, unmixing breaks down the spectrum of each pixel into a linear combination of pure component spectra (endmembers) and estimates the fractional abundance of each component. In tissue samples, these endmembers represent biochemical constituents such as hemoglobin, lipid, and water whose concentrations provide direct physiological insights without the need for labeled training data. According to the literature, there are two primary groups, which Table 8 summarizes.

**Physics-based unmixing:** By using the modified Beer–Lambert law, physics-based unmixing gets quantitative biomarker concentrations from reflectance spectra. Nardini et al. and Ricci et al. used the HyperProbe 1.1 to make oxygenation maps and NADH/FAD ratios for metabolic profiling of gliomas and meningiomas, respectively [60,61]. Clinicians can compare these values to known reference values, which makes this technique the clearest analytical method discussed in the review.

**Blind linear unmixing**: In contrast, blind linear unmixing determines the end members and their respective concentrations directly from the data without prior knowledge of tissue spectra. Cruz-Guerrero et al. showed that EBEAE achieved accuracy comparable to SVM while requiring less processing time [21]. Its real-time capability also demonstrated its potential for clinical application [62].

**Figure 10:**
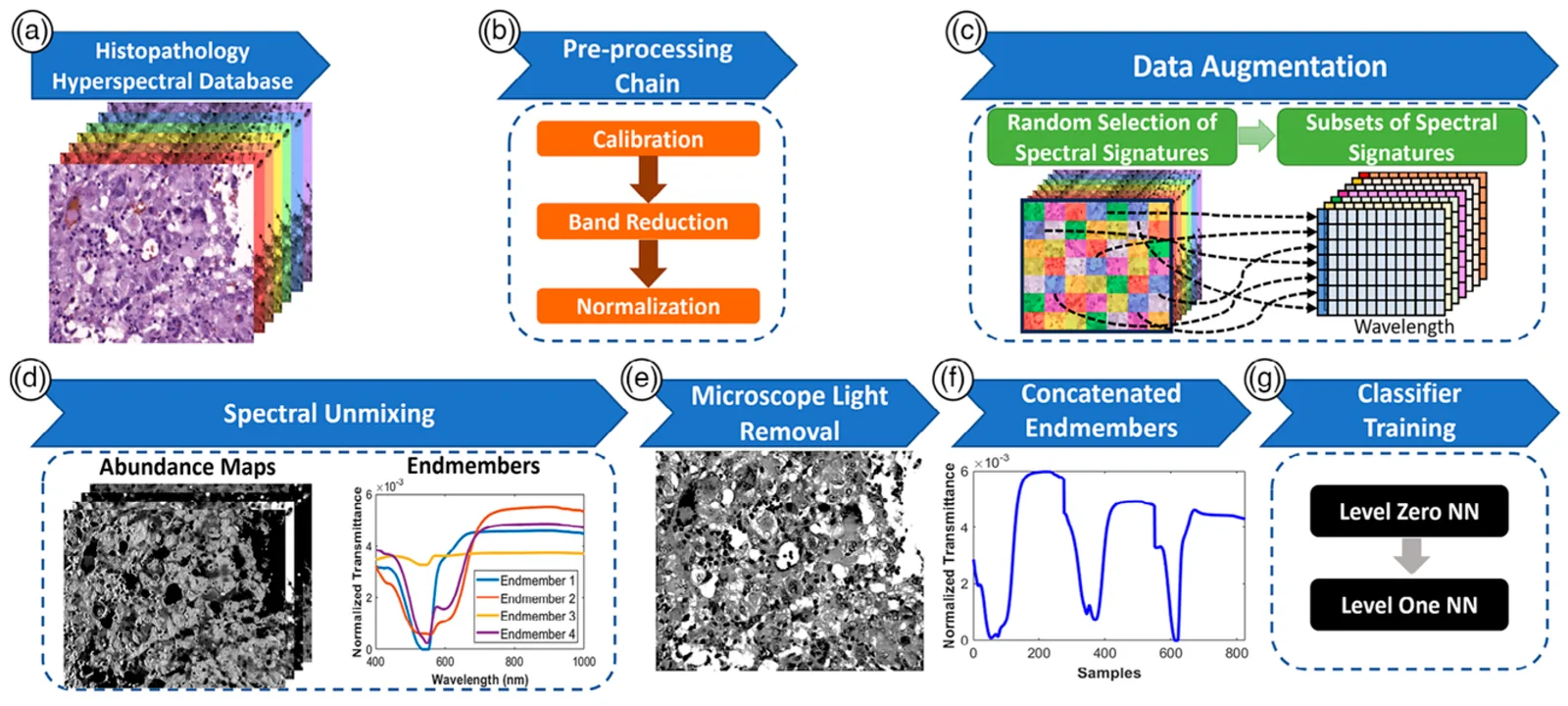
Overview of the hybrid hyperspectral imaging (HSI) unmixing and classification framework proposed by Cruz−Guerrero et al. (**a**) Histopathology hyperspectral database; (**b**) preprocessing, including calibration, band reduction, and normalization; (**c**) data augmentation through random selection of spectral signatures and generation of spectral subsets; (**d**) spectral unmixing to obtain pixel-wise abundance maps and endmember spectra representing distinct tissue components; (**e**) removal of microscope illumination artifacts from the extracted spectra; (**f**) concatenation of the corrected endmember spectra; and (**g**) two-level neural network classifier, in which the Level Zero network generates initial predictions and the Level One network refines the classification output. The different colors in the spectral plots represent distinct endmember signatures, whereas the colors in the HSI image stacks illustrate spectral bands [62].

The clinical value of unmixing extends beyond classification accuracy. Jong et al. showed that unmixing-based label assignment improved breast lumpectomy classification compared with center-pixel and patch-average methods [63]. Cruz-Guerrero et al. further combined EBEAE-derived endmember spectra with a deep neural network ensemble (Figure 10), being the only study in this review to explicitly integrate physics-based unmixing with learned classification [62].

Figure 10 presents the pipeline of the hybrid HSI framework. Starting from a histopathology hyperspectral database (a), the data undergoes preprocessing including calibration, band reduction, and normalization (b), followed by data augmentation through random spectral signature selection (c). Spectral unmixing then decomposes each pixel into abundance maps and endmember signatures representing distinct tissue components (d), after which microscope illumination artifacts are removed from the extracted spectra (e). The cleaned endmembers are concatenated (f) and passed to a two-level neural network classifier, where a Level Zero network performs initial classification and a Level One network refines the output (g). Together, these stages form a structured pipeline from raw spectral acquisition to tissue classification.

A common challenge faced by both approaches is the endmember selection issue. Physics-based techniques necessitate prior knowledge of tissue component spectra, which can differ among patients, devices, and preparation conditions. Blind methods, on the other hand, derive endmembers from the data but are influenced by the presumed number of components and the non-linear spectral mixing that linear models fail to address. While both challenges are surmountable, medical hyperspectral imaging has focused on them significantly less systematically than remote sensing, where unmixing techniques are notably more advanced.

**Table 8:** Summary of spectral unmixing techniques mainly covered in this literature.

Clinical Domain (Tissue Classification)	Method/Algorithm	Remarks/Best Performance	Key Limitation/Gap	Reference
Oncology—General Tissue	EBEAE-blind linear spectral unmixing	Comparable accuracy to SVM with ~400× faster computation	Small cohort and limited labeled tumor regions	[21]
Oncology—Brain/Neuro	EBEAE spectral unmixing + transfer-learning deep neural network ensemble	Accuracy: 88% ± 7%; F1: 92% ± 6%; Specificity: 80% ± 16%	Small dataset (13 patients) with imbalanced class representation	[62]
Breast Oncology	Spectral unmixing-based labeling + downstream classifier	Improved classification accuracy vs. center-pixel labeling	Limited differentiation between carcinoma subtypes	[63]
Oncology—Brain/Neuro	Physics-based spectral unmixing	Physiological biomarker maps	Small and unbalanced cohort	[61]
Oncology—Brain/Neuro	Physics-based spectral unmixing	Quantitative metabolic discrimination of tumor tissue	Proof-of-concept with a limited sample size	[60]

Recent research indicates that a deep learning-based hybrid framework allows models to comprehensively grasp spectral and spatial features through joint learning methods, even without the need for specific band selection or unmixing techniques [64]. This approach is explicitly tied to the model and its application in medical tasks, and the following section will discuss it in conjunction with task formulation.

## 6. Task Formulation in Medical HSI

One area of the medical HSI literature that receives surprisingly little explicit attention is the formulation of the clinical problem as a machine learning task. Choosing between classification, segmentation, detection, regression, or generation is not just a technical decision. It determines the requisite ground truth, the relevant performance metric, and ultimately, the applicability of the model’s output in a clinical setting. Table 9 compares the architecture and models used with formulated tasks in clinical HSI literature by looking at their annotation needs, learning strategies, and validation performance. The review of 130 studies found six main task formulations, each of which has its own clinical implications and limitations.

### 6.1. Pixel-Wise Classification

Pixel-wise classification assigns each spatial pixel in the HSI cube to one of a set of tissue classes that have already been defined [11]. These classes typically encompass tumor versus normal classifications or a multiclass framework that incorporates stroma, necrosis, vasculature, and infiltration zones. It appears in most brain, head and neck, and GI oncology studies, making it the most prevalent task formulation in the reviewed literature.

The appeal is simple to understand. The false-color map that results from each pixel producing a diagnostic label can be superimposed directly onto the surgical field. Using patch-based pixel classification on head and neck surgical specimens, Halicek et al. created tissue maps for 102 patients [1]. Fabelo et al. implemented a hybrid SVM and hierarchical clustering approach to show pixel-wise glioblastoma delineation in five intraoperative cases [37]. Wang et al. achieved an AUC of 0.950 for pixel-wise hepatocellular carcinoma classification using a 1D CNN with weighted loss [9].

The limitation of pixel-wise classification without spatial context is that adjacent pixels are treated as independent observations, ignoring the spatial continuity of tissue structure. This results in salt-and-pepper noise in classification maps. This is almost useless without post-processing and could lead to wrong conclusions in a clinical setting because of isolated misclassified pixels scattered throughout regions that are correctly labeled. Some studies use morphological smoothing, conditional random fields, or majority voting to deal with this, but these changes are made after classification instead of being part of the model.

### 6.2. Semantic Segmentation

Semantic segmentation explicitly models spatial context, or the relationship between each pixel and its neighborhood, while giving each pixel a class label. In medical HSI, this typically means delineating tumor boundaries, organ margins, or tissue compartments as spatially coherent regions rather than independent point classifications.

U-Net and its variants have become the standard segmentation architecture in the reviewed literature. Trajanovski et al. utilized channel selection and enhanced spatial context via U-Net to segment tongue squamous cell carcinoma, achieving strong performance (Dice ≈ 0.89, AUC ≈ 0.92) with limited data [65]. Seidlitz et al. employed a robust multi-organ segmentation across 20 tissue classes in open porcine surgery using U-Net, establishing the architecture’s intraoperative utility [66]. Bali et al. applied U-Net, UwU-Net, and a U-Net Transformer to head and neck tumor margin delineation, achieving 98% accuracy with histopathologically grounded ground truth [13]. Tang et al. introduced DeepLabV3+ with a convolutional triplet attention module for ovarian cancer segmentation, combining spatial attention with histopathology-guided voting to improve boundary precision [67].

The clinical benefit of segmentation compared to pixel classification is its interpretability; a smooth, spatially coherent tumor boundary map is significantly more actionable for a surgeon than a noisy pixel probability map. The corresponding cost is the annotation burden. Pixel-level ground truth masks are necessary for segmentation models, and each training specimen must be delineated by a pathologist [68]. This process is laborious, prone to inter-annotator variability, and intrinsically constrained by the two-dimensional nature of histological section annotation when the clinical problem is three-dimensional.

### 6.3. Instance Detection and Object Localization

A smaller group of studies defines the HSI task as object detection, which finds specific structures of interest in the image instead of sorting through every pixel. This formulation is most frequently used in dermatological and endoscopic applications where it is necessary to locate and identify discrete lesions within a larger field. Huang et al. approached lesion detection as an object detection problem and employed YOLOv5 for hyperspectral skin cancer classification [69]. Chen et al. compared detection architectures with whole-image classification techniques using YOLOv5, YOLOv8, and SSD for SAVE-reconstructed endoscopic HSI for esophageal cancer. The benefit of detection formulations in endoscopy is direct clinical alignment; endoscopists already report findings in the form of a bounding box surrounding a suspicious lesion [58]. The drawback is that bounding boxes lose the margin precision that distinguishes HSI from RGB endoscopy in a clinical setting.

### 6.4. Regression and Quantitative Biomarker Mapping

Instead of using discrete class labels, regression formulations predict continuous-valued tissue parameters from spectral data. This refers to measuring tissue water content, oxygenation (StO_2_), hemoglobin concentration, or metabolic markers throughout the imaged field in medical HSI. This task type is dominant in the perfusion and wound assessment literature.

Dietrich et al. measured microcirculatory oxygenation in patients undergoing heart surgery using direct spectral-parameter mapping without the need for a classification step [28]. Kohnke et al. combined automated ROI detection with Random Forest regression to use continuous spectral features to differentiate between sepsis and healthy tissue [18]. Nardinin et al. used the HyperProbe 1.1 to map the concentrations of hemoglobin and cytochrome c oxidase in real time, creating quantitative molecular maps instead of binary tissue labels [60].

Quantitative biomarker regression is probably the most clinically important task formulation because it gives results that are easy to understand in terms of physiology without requiring a clinician to use an algorithmic classification that they cannot check for themselves. The problem is ground truth: co-registered biochemical reference measurements are necessary for validating quantitative spectral biomarker estimates, but they are difficult to obtain and infrequently available in surgical settings.

### 6.5. Generative Tasks: Synthesis and Super-Resolution

A small but expanding group of studies synthesizes or reconstructs spectral data using generative models instead of classifying tissue. Ma et al. employed an unsupervised CNN to reconstruct high-resolution HSI from co-registered low-resolution HSI and RGB images, utilizing a super-resolution technique that enhances subsequent classification performance while reducing acquisition time [70]. This was further improved by adding a GAN-based method to make digital histological images from HSI data [71]. Subsequently, they created a pipeline to adapt hyperspectral imaging in digital pathology to support automatic histology diagnosis [72]. To facilitate a direct comparison with traditional endoscopic modalities, Yang et al. used CycleGAN to simulate narrow-band imaging from HSI. For cross-dataset generalization [73]. Hao et al. employed a hybrid CNN-LSTM-Transformer with domain adaptation. This task is partially generative since it entails learning mappings between spectral domains [74].

In the reviewed literature, generative approaches have the potential to be the most transformative task formulation, despite being the least clinically mature. If spectral synthesis from RGB can be proven, as Chen et al. try to do with SAVE, the whole hardware barrier to HSI adoption changes. The question is whether the synthesized spectral information retains enough biochemical fidelity for diagnostic decisions, not whether synthesis functions under controlled conditions [58]. There is currently no validation at the level needed for clinical implementation.

### 6.6. Multi-Task Learning

Several recent papers combine multiple task formulations within a single model. Kopriva et al. jointly performed classification and spectral feature extraction using a Grassmann manifold approach [42]. Li et al. employed GICAF-Net for concurrent graph-based spectral modeling and image-based classification in liver tumor diagnosis [75]. Yan et al. showed that annotation refinement, which is basically a pre-segmentation task, made the classification accuracy in lung cancer pathology go from 77.33% to 92.52% [76].

Multi-task learning is becoming a good way to solve the problem of not having enough annotations. If a model learns to segment tissue boundaries and classify tissue types at the same time, each task can help the other by giving it a supervisory signal. This means both tasks need less labeled data. This has a direct bearing on the issue of translational readiness, despite the fact that it is still under investigation and not yet a proven clinical approach. Because it implies that task architecture, instead of merely acquiring additional data, likely alleviates the data bottleneck.

Understanding clinical HSI research requires familiarity with both the data it relies on and the methods used to analyze it. Section 3 and Section 4 address the data foundation: imaging acquisition architectures determine how spectral cubes are captured across different clinical settings, while publicly available datasets define the benchmarks against which algorithms are evaluated and compared. Without standardized acquisition and accessible data, reproducible progress across the field is not possible. Section 5 and Section 6 address how this data is handled analytically: preprocessing and band selection reduce spectral dimensionality to what is clinically relevant, spectral unmixing separates overlapping tissue signals, and task formulation defines whether the clinical objective is tissue classification, segmentation, quantitative biomarker mapping, or lesion detection. These task types are not distributed uniformly—their selection depends on the clinical information required, the available annotations, and the intended role of the HSI output in the workflow. Section 7 examines how these elements come together across nine clinical domains and evaluates how far each has progressed toward clinical integration.

**Table 9:** AI architecture/model comparison by ML/DL task types with key details.

Architecture/Model	ML/DL Task	Spectral Learning	Spatial Learning	Labeled Data Required	Inference Speed	* OR-Compatible	Best Intra-Patient Accuracy	Best Inter-Patient Accuracy	Key Limitation	Reference
SVM + PCA/LDA	Detection/Classification	Linear only	None	Low	Very Fast	Yes	~85%	~75%	Cannot model non-lineal spectral–spatial tissue signatures; performance ceiling below clinical requirements	[37,52,77]
Spectral unmixing (NMF/EBEAE)	Classification	Endmember-based	None	None (unsupervised)	Very Fast	Yes	~88%	~76%	Assumes linear mixing of endmembers; struggles with spectral variability in vivo; no spatial context	[21,62,63]
U-Net	Segmentation	Selected bands	Pixel-wise	Moderate	Moderate	Partial	~93%	~84%	Requires pixel-level annotation, which is labor-intensive; spatial context without spectral depth	[47,67,78]
1D CNN	Diagnosis/Classification/Prediction	Full spectrum	Moderate	Moderate	Very Fast	Yes	~97%	~85%	No spatial context; pixel-wise only; misses margin topology and tissue boundary information	[79,80,81]
2D CNN	Detection/Classification/Diagnosis	Selected bands only	Full spatial	Moderate	Fast	Yes	~92%	~82%	Discards spectral depth; band selection introduces pre-classification bias	[82,83,84]
3D CNN	Classification/Prediction	Full spectrum	Full spatial	High	Moderate	Partial	~97%	~86%	High computational cost; requires large annotated datasets; inference too slow for most real-time OR settings	[85,86,87]
HybridSN/CNN (3D + 2D)	Classification	Full spectrum	Full spatial	High	Moderate	Partial	~97%	~87%	Training is data-intensive; computational cost is still high; validation is predominantly on benchmark datasets	[74,88,89]
Graph Neural Network (GNN)	Classification/Diagnosis	Node spectral features	Graph topology	High	Moderate	Not yet	~97%	~94%	Irregular graph construction adds complexity; limited to small clinical cohorts; no OR deployment evidence	[14,75]
Vision Transformer (ViT/Swin)	Segmentation	Global attention	Global attention	Very High	Low	Not yet	~98%	~88%	Requires large labeled datasets; slow inference on current surgical hardware; not yet OR-deployed	[39,90]
Universal Feature Perception Framework (UFPF)	Classification/segmentation	Joint learning	Joint learning	High	Low	Not yet	~87%	Not reported	Consistently outperformed (ResNet, ViT, Swin, FDNet, SpecTr); not cross validated	[64]
Unsupervised learning (MedDiff HSI)	Classification	Joint learning	Joint learning	No	Fast	Potential	98.1%	Not yet validated	Empirically selected hyper parameters; trained and tested on limited subset; no inter-patient or clinical validation	[91]
Semi supervised/ Self-supervised	Classification/Prediction	Learned from unlabeled data	Learned	Minimal labels	Moderate	Not yet	~97%	~92%	Pre-training domain mismatch; cross-site generalization not yet demonstrated at scale	[68,74,92]

* OR-compatible = inference achievable within 30 s on current surgical hardware. Inter-patient accuracy figures reflect leave-one-patient-out or independent test set validation only.

## 7. Clinical Applications Through Tier

The increasing integration of HSI-AI has expanded its potential across diverse clinical applications. This section synthesizes current evidence across major medical domains, focusing on the progression from technical validation to clinical implementation. Studies are considered in terms of AI-based analysis, cohort scale, patient-level or external validation, workflow integration, and clinical readiness, with applications categorized according to their level of translational development.

### 7.1. Brain and Neuro-Oncology

**Figure 11:**
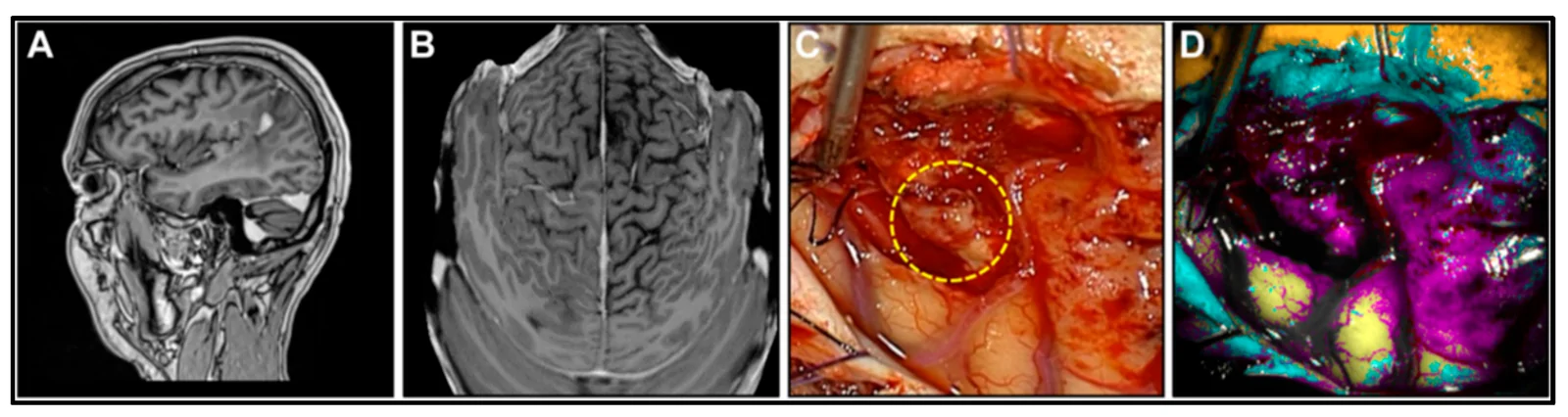
Workup for HSI-guided intra-axial surgery. Adapted from Puustinen et al. [95]. Preoperative MRI reconstructions (**A**) and cortical surface maps (**B**) support surgical planning but may not reveal microscopic tumor regions, particularly in previously operated or irradiated tissues ((**C**), yellow circle). HSI classification maps (**D**) can help identify and localize otherwise difficult-to-distinguish tumor islands (violet) during reoperation.

Glioblastoma infiltration can extend beyond visible tumor margins, while conventional white-light imaging [37] and 5-ALA fluorescence have limitations in detecting infiltrative regions [44]. Recent studies have increasingly integrated HSI with artificial intelligence for automated brain tumor analysis [45]. Machine-learning classifiers have been used to distinguish tumor margins, WHO grades, tissue types, and IDH status from spectrally derived information [93], while SAEDCNN combined labeled and pseudo-labeled HSI data for brain tumor detection with reduced dependence on extensive annotations [85]. More recent approaches, including MedDiffHSI and KAN-WAL Net, have applied spectral–spatial diffusion learning and computationally efficient deep-learning architectures, respectively, for brain tissue and tumor classification [90,91]. Clinically integrated HSI hardware support its classification within Tier 2 clinical readiness [94]. Importantly, Puustinen et al. proposed (Figure 11) a practical clinical framework and standardization strategy for integrating HSI and machine learning into intra-axial brain tumor surgery, emphasizing the need for larger shared datasets and stronger clinical evidence before widespread adoption [95].

Figure 11 illustrates how HSI can complement conventional neurosurgical planning by adding intraoperative tissue information to preoperative MRI (A) and cortical surface maps (B). Microscopic tumor regions that may not be clearly distinguishable from surrounding tissue, particularly during reoperation (C), which is clearly identified by HSI classification maps (D) (violet color show the tumor islands).

### 7.2. Head and Neck Oncology

**Figure 12:**
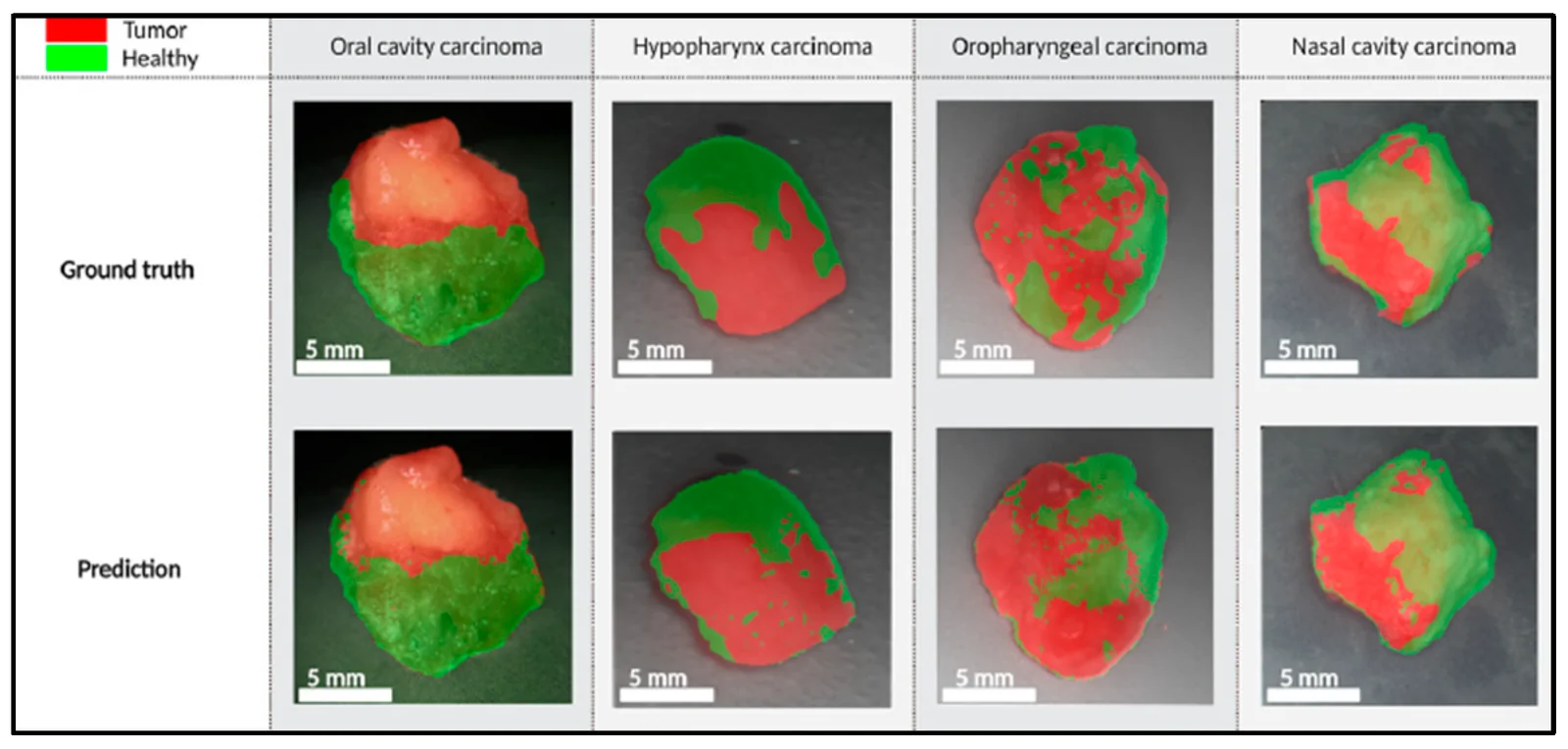
AI-based segmentation for HNSCC samples across diverse tumor entities. Adapted from Bali et al. [98].

HSI has been increasingly investigated for tumor detection and margin assessment in head and neck oncology [11]. Early studies explored spectral differences between tumor and surrounding tissue, while more recent work has integrated machine learning and deep learning for automated analysis [65]. Maktabi et al. demonstrated that HSI combined with a multilayer perceptron could distinguish tumor from squamous epithelium in esophageal specimens [96]. More recent studies have expanded HSI analysis to hyperspectral histology, including an automated processing framework and benchmark dataset containing more than 350 head and neck cancer slides [72]. Experiments using polarized HSI have also been explored in automated pathological assessment [97]. Bali et al. subsequently demonstrated an intraoperative workflow combining HSI, DL, and 3D modeling for rapid, label-free tumor-margin assessment in HNSCC (Figure 12) [98]. Their subsequent CE-certified and MDR-compliant study further investigated the integration of HSI into head and neck surgery using classical machine-learning approaches [13]. Other recent work has explored HSI analysis of laryngeal and pharyngeal mucosa using 3D spatio-spectral deep learning [99], as well as HSI-based prediction of treatment response in esophageal cancer [88]. Together, these studies show continued movement from experimental tissue characterization toward automated tumor assessment [100] and clinically integrated HSI workflows, supporting the classification of head and neck oncology as a Tier 2 readiness.

Figure 12 illustrates the CNN-based tumor predictions alongside corresponding ground truth annotations across multiple tumor entities, including oral cavity, hypopharynx, and oropharyngeal carcinomas. The figure showcases the automated segmentation maps for excised tissue specimens generated by the CNN model trained on hyperspectral imaging data. By providing precise, binary delineation between healthy tissue and residual tumor within a clinically viable timeframe, the model underscores the translational potential of this system as a real-time intraoperative decision-support tool.

### 7.3. GI, Colorectal and Hepatobiliary Oncology

**Figure 13:**
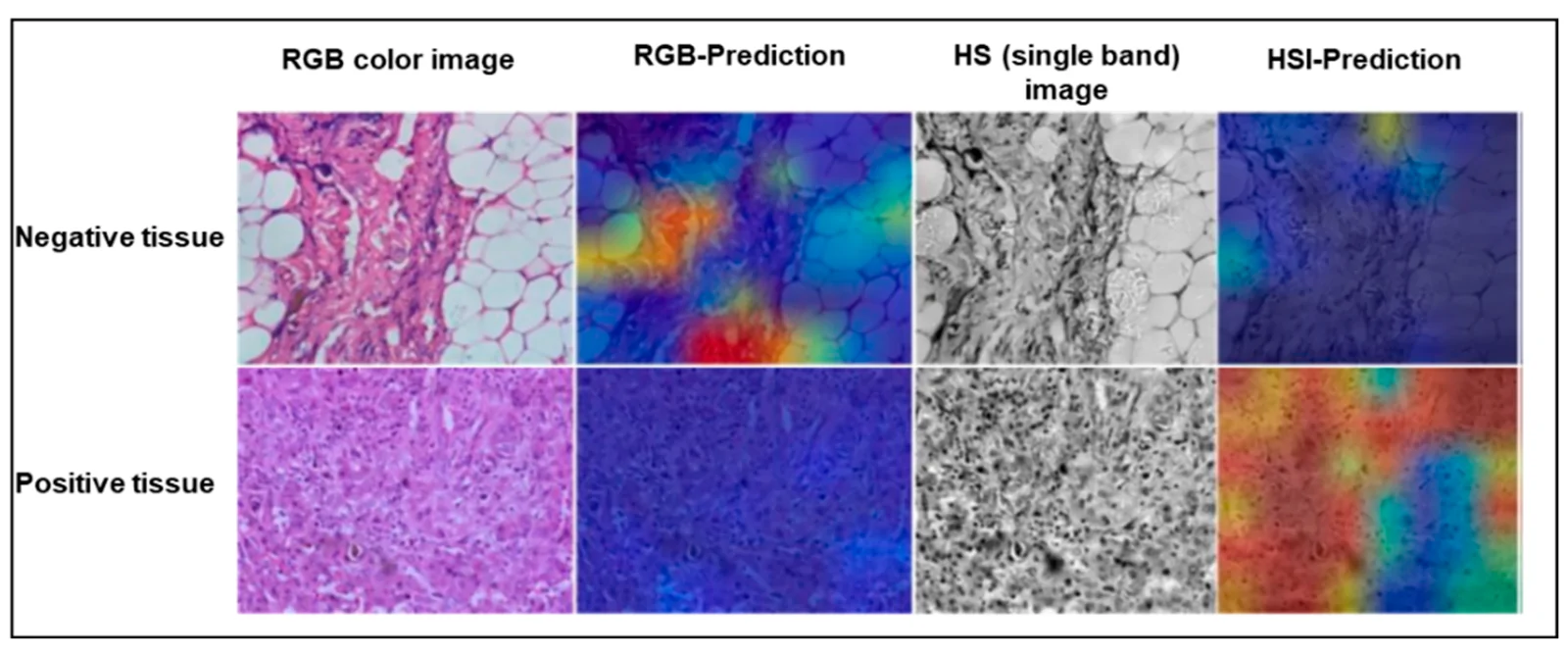
CNN-based predictions for cholangiocarcinoma using microscopic hyperspectral imaging proposed by Sun et al. Red indicates high confidence for tumor, whereas blue indicates high confidence for non-tumor tissue. All images were acquired at 20× magnification. The top row shows a negative slide correctly classified by both RGB and hyperspectral image-based CNN models, while the bottom row shows a positive slide in which the RGB-based model fails to detect the tumor, whereas the hyperspectral model identifies the suspicious tumor regions, as verified by an experienced pathologist. The third column shows a representative single spectral band from the hyperspectral image cube [40].

HSI has been investigated across colorectal, gastric, hepatic, and biliary cancers, with applications ranging from tissue differentiation to intraoperative tumor assessment [101]. Baltussen et al. demonstrated that HSI could distinguish fat, healthy colorectal wall, and tumor tissue, supporting its potential for intraoperative guidance [77]. More recent studies have incorporated deep learning and larger clinical datasets. Kumar et al. evaluated a customized 3D U-Net for cholangiocarcinoma using data from 174 patients and reported improved performance compared with existing approaches [78]. Figure 13 illustrates microscopic hyperspectral classification of cholangiocarcinoma proposed by Sun et al. [40]. Cinar et al. showed that hyperspectral microscopy combined with a 3D CNN could classify H&E-stained hepatocellular carcinoma tissue with higher accuracy than 2D CNN and RGB-based approaches [80,84]. Other recent work has explored 3D CNN-based colorectal cancer detection [51], improved deep residual networks for gastric cancer differentiation [102], and graph-based spectral-structural learning for intraoperative differentiation of focal nodular hyperplasia and hepatocellular carcinoma [75]. These studies indicate continued expansion of HSI-AI applications across gastrointestinal and hepatobiliary oncology, although the level of clinical validation varies between applications. This domain is therefore classified as Tier 2.

Figure 13 shows how microscopic hyperspectral imaging (MHSI) outperforms standard RGB images when a CNN model detects cholangiocarcinoma. In the top row, standard RGB imaging relies only on broad color patterns, leading the model to misidentify healthy fibrous tissue as a tumor (false positive, red/yellow in column 2). When the model uses the full HSI spectral cube instead, it identifies the tissue’s true biochemical signature and correctly marks it as non-tumor (blue in column 4). In the bottom row, standard RGB imaging lacks enough contrast to spot malignant cells, causing the model to completely miss the tumor (false negative, blue in column 2). In contrast, microscopic HSI captures continuous narrow wavelength bands that highlight sub-visual cellular changes. This allows the HSI-trained model to accurately pinpoint the tumor region with high sensitivity (red in column 4), demonstrating that HSI can improve cholangiocarcinoma detection.

### 7.4. Intraoperative Guidance for Multi-Organ

**Figure 14:**
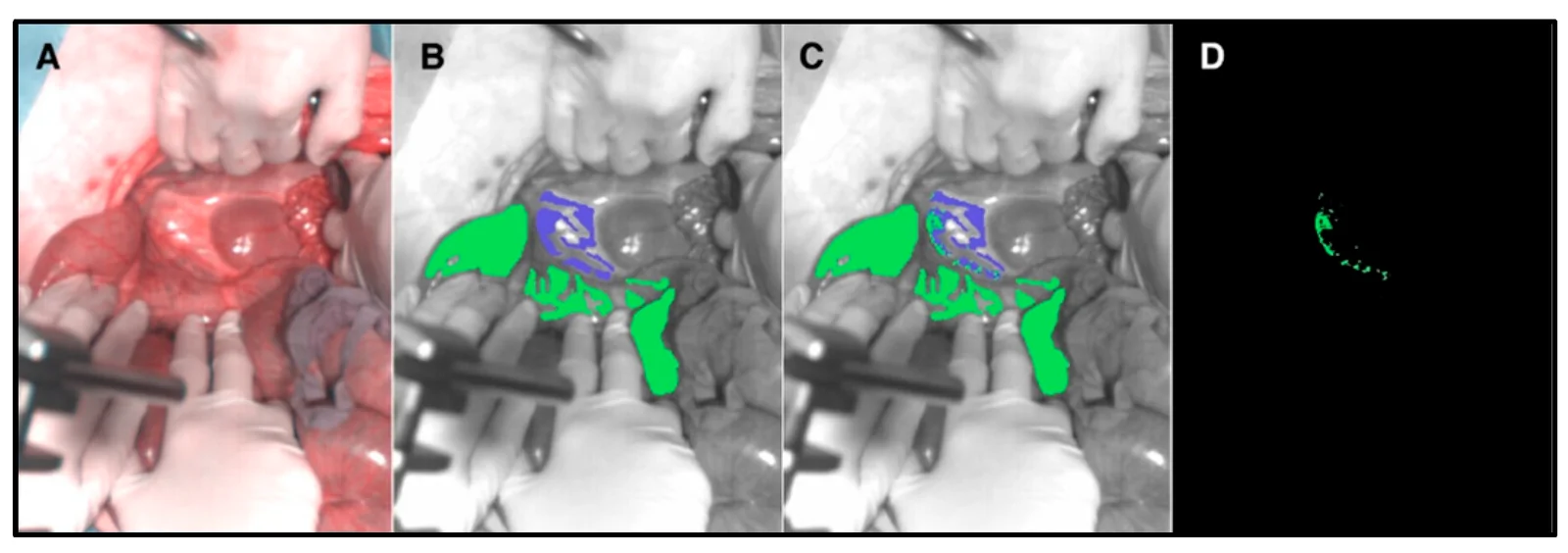
Real-time AI-assisted hyperspectral guidance for multi-organ surgical navigation and pixel-level tissue boundary delineation. (**A**) Standard RGB image extracted from the HSI cube; (**B**) expert-provided ground-truth annotation, with blue indicating retroperitoneal tissue (to be preserved) and green indicating colon–mesocolon tissue (to be resected); (**C**) CNN-generated pixel-wise tissue predictions; and (**D**) error map highlighting misclassified pixels to illustrate the differences between the model predictions and expert annotations. Adapted from Okamato et al. [32].

Beyond individual cancer types, HSI has also been explored as a general intraoperative tool for identifying multiple tissue and organ types. Studier-Fischer et al. demonstrated automatic discrimination of 20 organ classes in vivo with more than 95% accuracy in a preclinical model [25]. The field has since progressed toward human studies. Bannone et al. conducted a prospective bi-center study in which HSI combined with CNN analysis enabled automatic recognition of 13 abdominal tissue categories in vivo (Figure 14), demonstrating the feasibility of real-time tissue identification during surgery [17]. Other studies have evaluated HSI for multi-organ segmentation in open surgery [32] and reported that raw hyperspectral data could outperform RGB and processed tissue-parameter images [66]. Studier-Fischer et al. further addressed technical aspects of image quality and annotation consistency through a dedicated surgical HSI dataset [19]. More recently, Sellner et al. investigated HSI data from 230 human patients together with porcine and rat models and showed that physiological augmentation could help address spectral differences between species [92]. Together, these studies show a shift from preclinical tissue recognition toward broader intraoperative validation and dataset development, supporting the classification of multi-organ guidance as a Tier 2 clinical readiness.

Figure 14 demonstrates how the trained CNN model performs automated tissue recognition on a representative hyperspectral image from the dataset (Panels A–D). Panel A displays the standard RGB image extracted directly from the HSI cube. Panel B shows the corresponding ground truth annotation provided by expert surgeons, where blue highlights retroperitoneal tissue (to be preserved) and green highlights colon–mesocolon tissue (to be resected). Panel C illustrates the CNN model’s automated pixel-wise tissue predictions within these annotated regions, showing strong visual agreement with the expert annotations. Finally, Panel D presents the error map, highlighting all misclassified pixels to provide a clear, pixel-level evaluation of model accuracy.

### 7.5. Wound Assessment and Perfusion Monitoring

**Figure 15:**
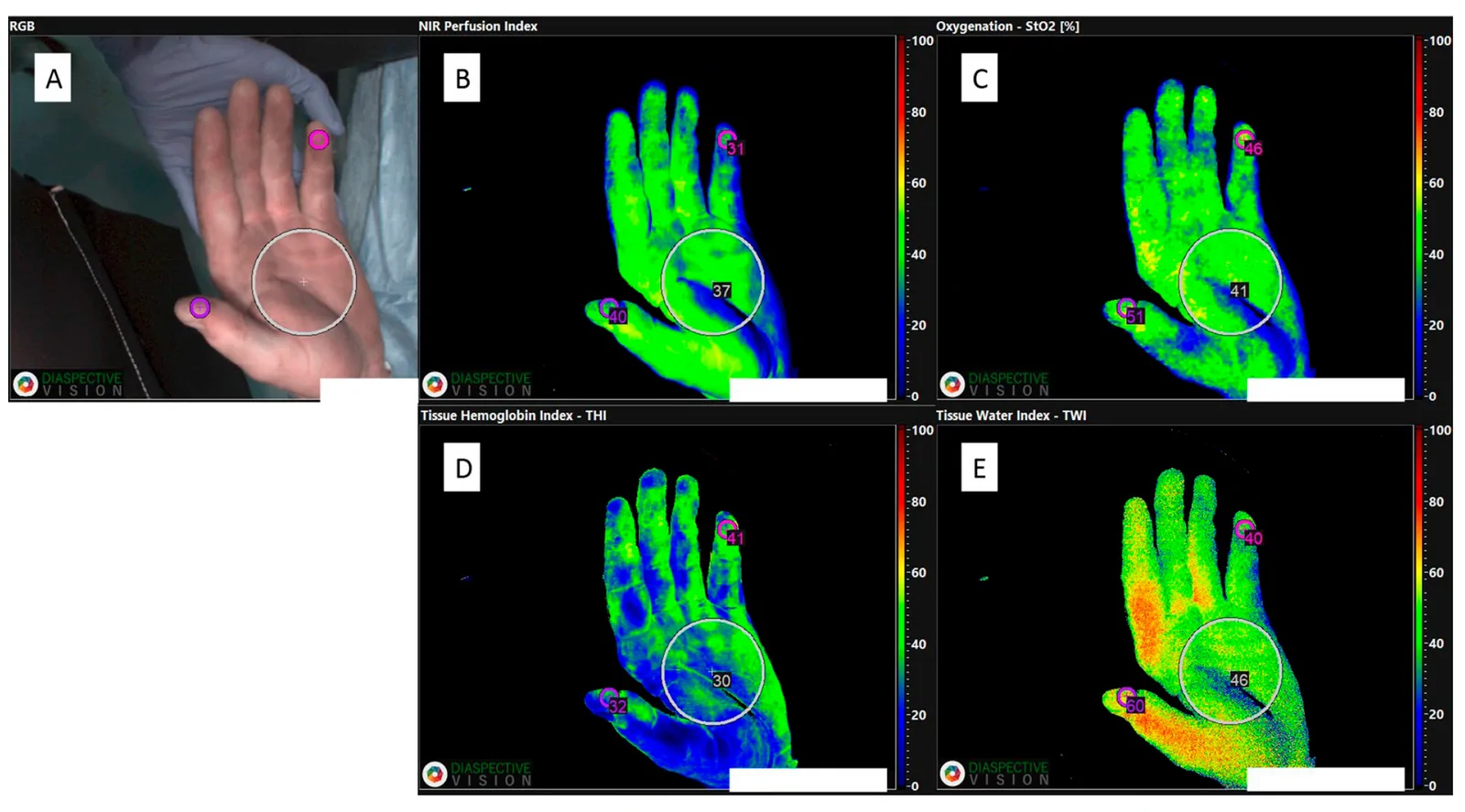
Microcirculatory impairment assessment by Katzenschlager et al. using HSI in a trauma resuscitation room [31]. (**A**) Standard RGB image of the patient’s hand showing the regions of interest (ROIs) across the palm and fingertips; (**B**) Near-Infrared Perfusion Index (NIR) map; (**C**) tissue oxygen saturation (StO_2_) map; (**D**) Tissue Hemoglobin Index (THI) map; and (**E**) Tissue Water Index (TWI) map. In the pseudo-color maps, red/yellow represents higher values (50–100): good-to-moderate perfusion, whereas green/blue indicates lower values (0–50): lower perfusion, providing label-free visualization of tissue perfusion and physiological status.

HSI-AI has also been applied to wound assessment and tissue perfusion, where spectral information can provide objective information about tissue oxygenation and vascular status. Cihan et al. used a 3D CNN to characterize neonatal cutaneous wounds and reported improved performance compared with a 2D CNN [89]. HSI has also been investigated for diabetic foot ulcer assessment using a hybrid CNN-SVM approach [103]. More recent work has extended these applications to intraoperative perfusion assessment. Shuplestov et al. combined HSI with machine learning to classify intestinal ischemia with more than 95% accuracy and demonstrated feasibility in pilot human cases, although the model was trained using animal data and requires validation in larger human cohorts [29]. Other clinical studies have used HSI to monitor abnormal fingertip oxygenation (Figure 15) and rewarming in patients with systemic sclerosis-associated Raynaud phenomenon [104] and to assess colon conduit perfusion during surgery [105]. These studies indicate that HSI is moving beyond tissue classification toward quantitative assessment of perfusion and physiological changes, while larger and clinically representative validation studies remain important for wider adoption. This domain is classified as Tier 2. Strong clinical evidence, including an RCT, positions this as the most mature non-oncological domain at Tier 2 [31].

Figure 15 showcases non-invasive microcirculatory assessment using a commercial HSI system during initial resuscitation in a trauma setting. Panel A provides the standard RGB orientation image of the patient’s hand, defining regional regions of interest (ROIs) across the palm and fingertips. Panels B through E display pseudo-color quantitative parameter maps generated from the hyperspectral cube, where red/yellow denote high numerical values (50–100) and green/blue represent reduced perfusion (0–50). Specifically, Panel C maps tissue oxygen saturation (StO_2_) while Panels B, D, and E map the Near-Infrared Perfusion Index (NIR), Tissue Hemoglobin Index (THI), and Tissue Water Index (TWI), respectively. This multi-parameter breakdown provides immediate, label-free physiological feedback on tissue ischemia and fluid shifts without requiring intravenous contrast agents.

### 7.6. Breast Oncology

**Figure 16:**
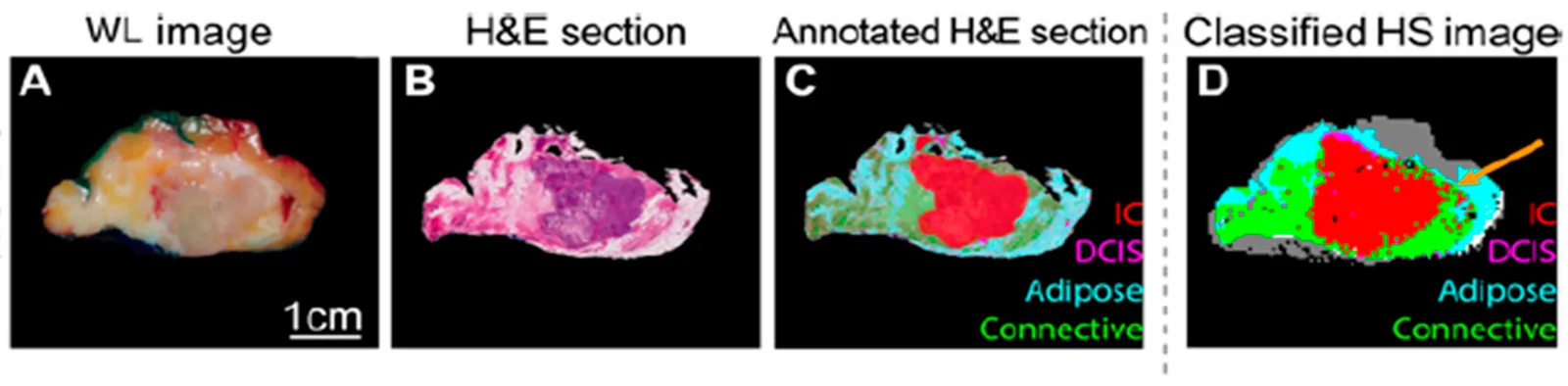
Hyperspectral analysis of the breast resection slices by Kho et al. (**A**) White-light image, (**B**) histopathology H&E sections, (**C**) annotated H&E sections, and (**D**) HS classification using the SVM algorithm [106].

In breast oncology, HSI has been investigated mainly for surgical margin assessment, tissue characterization, and postoperative complications. Kho et al. provided an early demonstration of HSI for breast-conserving surgery and showed that HSI combined with SVM could identify 19 of 20 malignancies located within 2 mm of the resection margin (Figure 16) [106]. Jong et al. later evaluated hyperspectral unmixing in 189 lumpectomy specimens and reported improved tissue classification compared with center-pixel and average-patch approaches [63]. Yu et al. investigated spectral features for differentiating granulomatous mastitis from normal breast tissue [107], while Quintana et al. explored multimodal HSI, whole-slide imaging, and clinical data for breast cancer recurrence prediction using a public dataset with long-term follow-up [41]. More recent studies have expanded the application of HSI beyond margin assessment. Hwang et al. used hyperspectral dark-field microscopy to detect and classify tissue types, including carcinoma, in post-lumpectomy breast tissue [108]. In a prospective multicenter study, Weiss et al. further demonstrated the potential of HSI-derived measures for objective assessment of breast cancer-related lymphedema [109]. These studies show that breast HSI is expanding from ex vivo margin assessment toward disease characterization, recurrence analysis, and postoperative monitoring, supporting its classification as a Tier 2 readiness level.

Figure 16 details ex vivo margin assessment on resected breast tissue slices. Panel A presents the macro white-light image of the gross tissue resection (Patient #5). Panels B and C display the corresponding histology and hematoxylin–eosin (H&E) section and its annotated gold-standard ground truth, delineating invasive carcinoma (IC, red), ductal carcinoma in situ (DCIS, pink), adipose tissue (cyan), and connective tissue (green). Panel D depicts the automated SVM classification map derived from the hyperspectral data cube. The classified HS image demonstrates high spatial concordance with histological annotations, successfully identifying central invasive tumor boundaries (red) and surrounding adipose margins (cyan) across whole-specimen cross-sections.

### 7.7. Gastroenterology and Endoscopy

**Figure 17:**
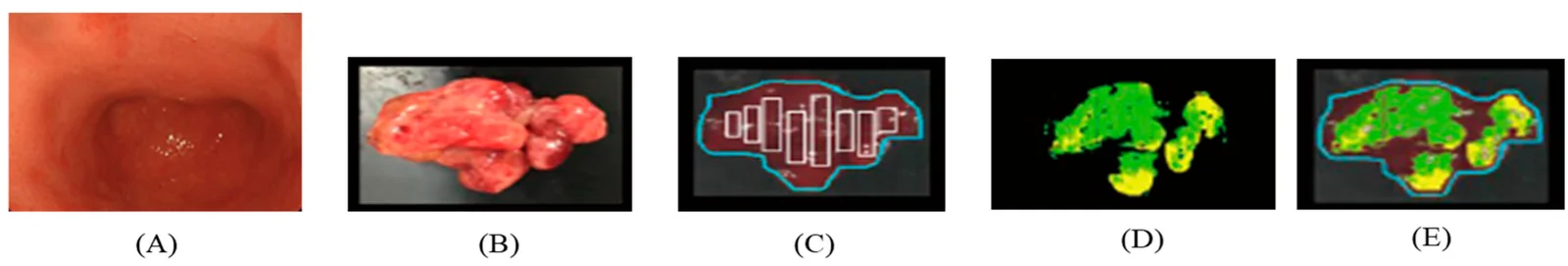
NIR-HSI analysis by Sato et al. (**A**) Endoscopic view of a submucosal tumor (SMT), not detectable by conventional endoscopy; (**B**) visible-light specimen image; (**C**) pathologist annotation (ground truth); (**D**) machine learning-based prediction; (**E**) corresponding annotation (blue boundary, yellow = normal; green = tumor) [30].

Early dysplasia in Barrett’s esophagus, gastrointestinal metaplasia, submucosal tumor lesions, and colorectal serrated lesions require biochemical differentiation at speeds compatible with standard endoscopic procedures [110]. Sato et al. demonstrated that near-infrared HSI combined with machine learning could distinguish gastric gastrointestinal stromal tumors from normal mucosa despite submucosal coverage (Figure 17), supporting its clinical feasibility in endoscopy [30]. Qin et al. subsequently developed an HSI-based CNN for pancreatic ductal adenocarcinoma cytology obtained through EUS-guided sampling and reported improved performance compared with an RGB-based CNN [36]. More recent work has investigated HSI-CNN analysis of pancreatic cytology for differentiating pancreatic ductal adenocarcinoma from pancreatic neuroendocrine tumors, with reported sensitivity and specificity above 90% and the use of visualization methods to support model interpretation [111]. These developments extend HSI from conventional tissue imaging toward endoscopic and cytological applications that may support minimally invasive diagnosis. However, recent reviews continue to identify the cost of HSI systems, technical complexity, and the lack of standardized acquisition and analysis protocols as barriers to broader clinical adoption [112]. Snapshot integration remains experimental, placing this domain at Tier 2.

Figure 17 highlights near-infrared hyperspectral imaging (NIR-HSI) for gastric submucosal tumors (SMTs). Panel A shows the in vivo endoscopic view, where the submucosal lesion remains hidden beneath visually intact mucosal tissue. Panel B presents the resected tissue specimen under visible light, and Panel C illustrates the pathologist’s ground truth annotation outlining the lesion boundary (blue line) and histological sampling regions. Panel D presents the machine-learning spectral classification map, distinguishing neoplastic regions (green) from healthy tissue (yellow), while Panel E overlays this prediction on the annotated specimen boundary. This demonstrates how NIR-HSI penetrates superficial layers to reveal deep biochemical signatures that are completely invisible during routine white-light endoscopy.

### 7.8. Dermatology

**Figure 18:**
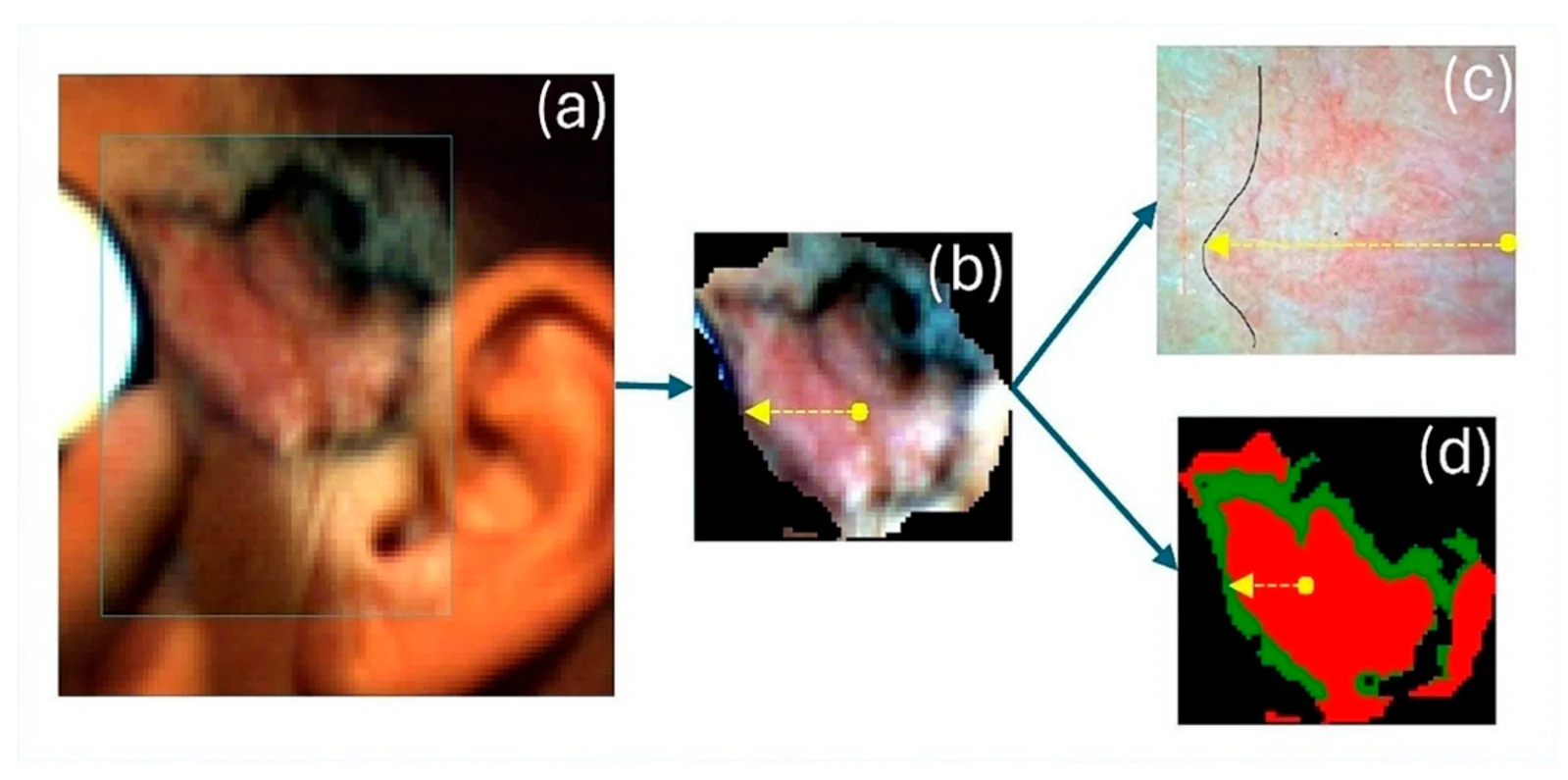
In vivo skin carcinoma margin assessment by Parasca et al. Maximum horizontal tumor distance used to compare the TMD method with dermoscopy [18]. (**a**) Original hyperspectral image of a basal cell carcinoma in the temporal/preauricular region of a 57-year-old woman; (**b**) region of interest showing the maximum horizontal distance (yellow arrow) from the tumor center to the anterior margin; (**c**) dermoscopic image highlighting the tumor margin at the anterior pole; and (**d**) classification map generated from hyperspectral data.

Dermoscopic evaluation cannot reliably distinguish all malignant subtypes without biopsy [34,69]. Parasca et al. used HSI with a spectral-angle-based classifier for in vivo assessment of basal cell carcinoma and squamous cell carcinoma margins (Figure 18) and reported AUC values above 0.89 [6]. Huang et al. applied an AI-HSI framework to distinguish mycosis fungoides from psoriasis and atopic dermatitis, demonstrating the potential of spectral information for differentiating clinically similar skin conditions [34]. Tian et al. used hyperspectral pathology images with a deep residual network and transfer learning to distinguish melanoma from nevus with 98% accuracy [83]. More recent work has expanded the range of dermatological applications, with VIS-NIR HSI enabling label-free detection of Sarcoptes scabiei mites in human skin tissue [113]. Overall, these studies demonstrate applications ranging from in vivo cancer-margin assessment to pathological diagnosis and label-free detection of infectious agents. However, small cohort sizes and lack of multicenter validation position this domain at Tier 2–3.

Figure 18 illustrates in vivo cutaneous basal cell carcinoma margin assessment. Panel a shows the clinical macroscopic view of the skin lesion located near the ear canal, while Panel b isolates the specific region of interest (ROI) and indicates the vector measuring the anterior tumor margin distance. Panel c displays the dermoscopic close-up highlighting the visually estimated tumor boundary (black curve). Panel d presents the classification map derived using the Three Maximum Density (TMD) method on hyperspectral data, where tumor regions are depicted in red. The TMD-based hyperspectral classification map delineates sub-clinical tumor extensions beyond what is visible under dermoscopy, offering a label-free approach to guide surgical resections.

### 7.9. Emerging and Exploratory Applications

Twenty-three papers address domains outside established oncological and perfusion categories: rheumatology and orthopedics [114,115], dental and oral medicine [116,117], respiratory pathology [118], renal pathology [119], coronary artery diagnosis [120], gynecologic oncology [67], and cardiac electrophysiology [14]. None of the emerging domains have attained inter-patient validation at a scale sufficient for translational claims, and all the studies are classified at Tier 3 (proof-of-concept, single-site, small cohort). Two areas stand out as immediate priorities. The ophthalmology cluster, which includes retinal HSI for Alzheimer’s disease and AMD biomarkers [10,33], has many potential patients, no invasive procedures, and existing imaging infrastructure. However, there is still not enough evidence. The gynecologic oncology papers show that coordinated multi-site studies are possible in the current workflows for colposcopy and cytoreductive surgery [121,122]. Furthermore, early studies on nanoscale drug delivery have taken place in the pharmaceutical field [123].

Using these levels of clinical evidence, the validation strategies mainly help with higher translation readiness, which will be discussed in more detail in the next section, along with a full domain-level appraisal that is summarized in Table 10.

## 8. Evaluation Frameworks and Translational Readiness

Evaluation protocols play a central role in determining translational readiness, as clinical deployment depends not only on model performance but also on the rigor and generalizability of validation strategies [124]. Numerous medical HSI studies indicate elevated accuracy and AUC values [125]; however, these findings frequently originate from single-center datasets employing internal cross-validation [126]. Translational readiness, however, requires patient-level independence, external validation, and clinically meaningful metrics that reflect diagnostic utility [112,127]. As a result, the way studies are assessed affects whether their reported results indicate that the idea could work in practice or if they are ready for use. None of the reviewed studies achieved the Tier 1 evidence required for clinical deployment. About 48.5% (63 papers) reached Tier 2 with strong single-site validation but lacked multicenter studies, while 50.8% (66 papers) remained Tier 3 proof-of-concept investigations. The randomized controlled trial by Katzenschlager provided the highest level of individual evidence but remained Tier 2 due to its single-center design [31].

**Figure 19:**
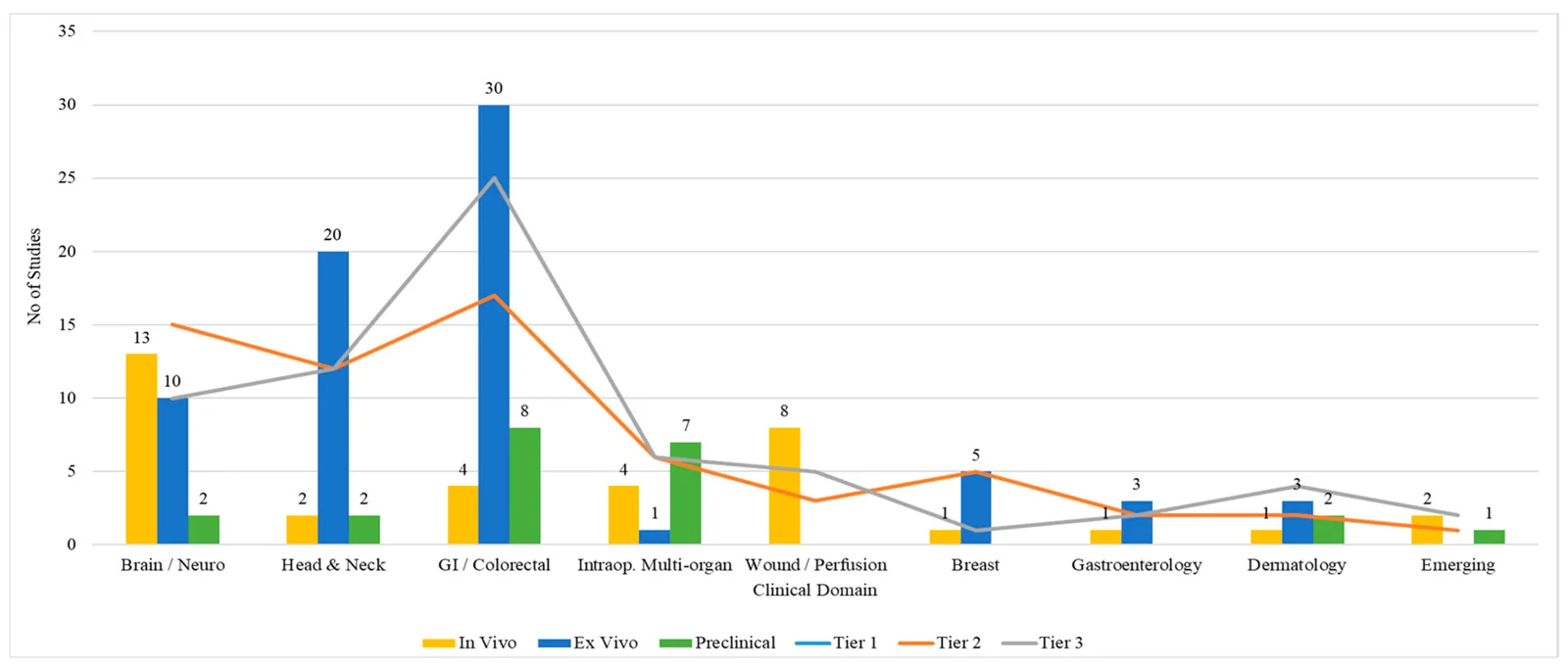
Distribution of 130 included studies across nine clinical domains, stratified by clinical context (stacked column) and translational readiness tier (line). Tier 1 = multicenter prospective evidence; Tier 2 = single-site inter-patient validation; Tier 3 = proof-of-concept or intra-patient validation only.

Figure 19 summarizes the distribution of 130 included studies across nine clinical domains, stratified by translational readiness tier and clinical context (in vivo, ex vivo, and preclinical). This distribution shows three main patterns. First, the literature is dominated by oncological applications, which constitute about 74% of studies. This indicates a strong alignment with the needs of surgical decision-making. Second, ex vivo research significantly exceeds in vivo research in the majority of fields, with brain and neuro-oncology being the main exceptions. This underscores the ongoing disconnect between laboratory validation and clinical implementation.

## 9. Translational Barriers: From Lab to Clinic

**Figure 20:**
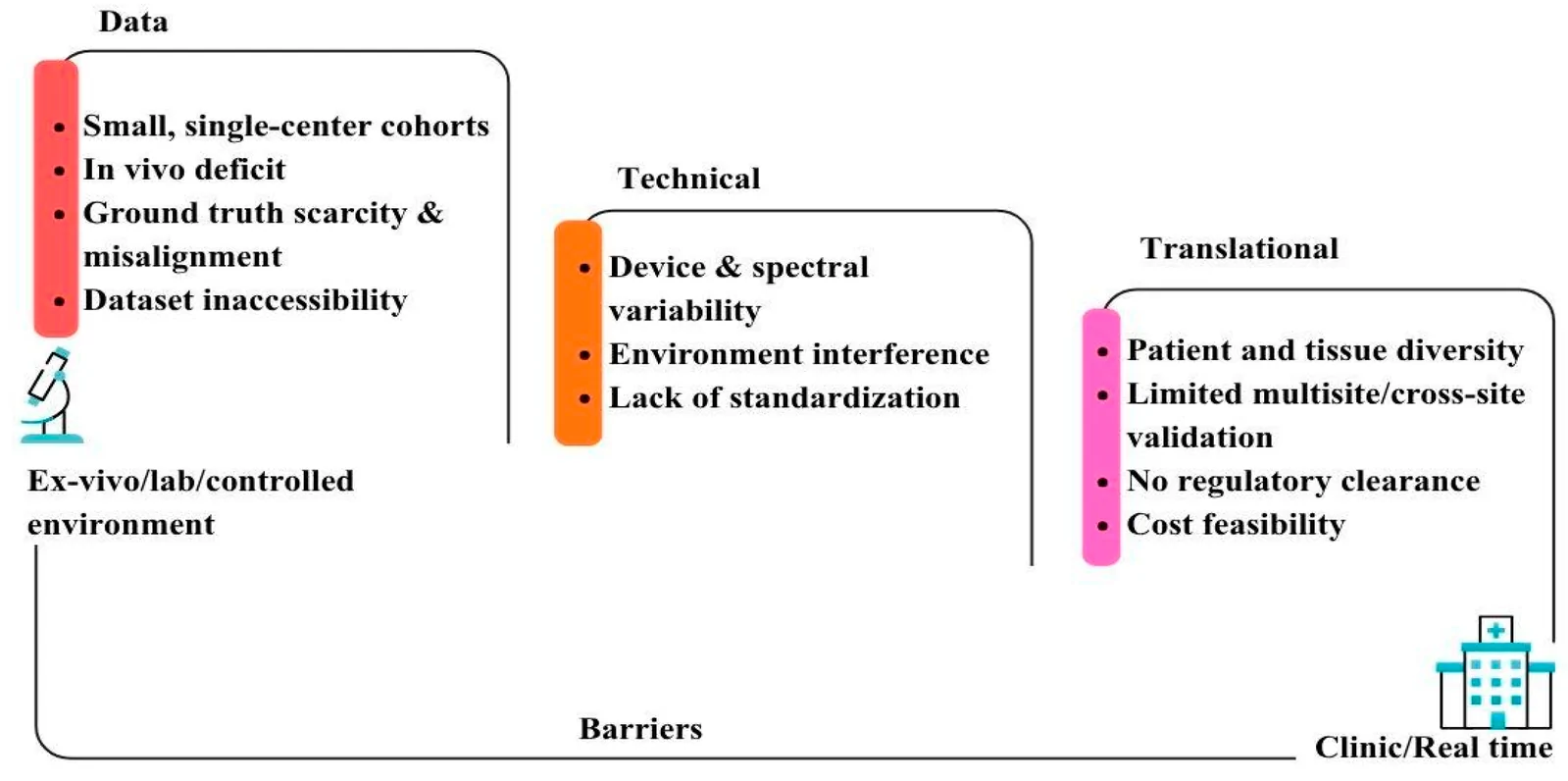
Illustration of key translational limitations of HSI from laboratory development to clinical deployment.

The tier distribution in Section 6 reflects not technical capability but the gap between demonstrated performance and clinical deployment. This gap arises from specific, addressable barriers that have received less attention than algorithmic and hardware advances (Figure 20). This section examines these barriers using limitations reported across all 130 reviewed studies.

### 9.1. Dataset Size and Validation Design

Of the 130 reviewed studies, 55 (42.3%) reported small sample sizes, increasing the risk of overfitting and limiting representation of inter-patient spectral variability. Only five studies have included multicenter data, which further exacerbates this problem and limits the generalizability of the model across clinical settings.

Validation design further amplifies this issue. Patch-based k-fold cross-validation (used in 51 studies, 39.2%) often lacks patient-level separation, causing intra-patient data leakage and inflated performance estimates. Studies with strict inter-patient or independent testing report lower, more realistic accuracies (~70–80%) [22,88], whereas 32 studies report >95%, highlighting a clear gap between reported and real-world performance [89].

### 9.2. Dataset Availability and In Vivo Deficit

Beyond validation design, the dataset landscape itself presents structural barriers to clinical translation. Ex vivo studies dominate the literature, particularly in oncological applications. For example, 85% of gastrointestinal and colorectal oncology studies and 79% of head and neck studies rely on ex vivo tissue imaging. These controlled experimental conditions differ substantially from intraoperative environments, where motion, blood pooling, illumination variability, and tissue heterogeneity affect spectral acquisition. Because of this, performance in ex vivo settings may not be the same as performance in real-life clinical settings.

Dataset accessibility further limits progress. Only 9 of 130 studies (~7%) provide publicly available datasets (Table 5), while 28 (21.5%) offer data upon request, and 65 (50%) provide no data availability statement. This lack of open data prevents replication, benchmarking, and multi-site validation, all of which are required for Tier 1 clinical evidence. Moreover, available datasets are concentrated in specific domains, particularly brain tumor imaging, where the HELICoiD dataset is repeatedly used across multiple studies. While beneficial for benchmarking, repeated use of the same dataset limits independent validation and may overestimate algorithmic generalizability.

### 9.3. Annotation and Ground Truth Limitations

Annotation represents another major translational bottleneck in medical HSI. Unlike conventional RGB imaging, hyperspectral data consist of high-dimensional spectral cubes requiring expert interpretation across both spatial and spectral domains. Consequently, most studies rely on pseudo-RGB projections or single-band visualizations for annotation, rather than direct spectral interpretation. This introduces a mismatch between the spectral features used by models and the visual cues used for annotation.

Ground truth quality presents an additional challenge. A total of 78 studies (60%) use histopathology as the gold standard, but histopathological labels are usually at the region level rather than the pixel level [70]. When such labels are used for pixel-wise classification, spatial uncertainty is introduced into the training data. A few studies have investigated this problem using precise spatial registration or three-dimensional histological reconstruction [128]. This underscores the necessity for improved methods to annotate and standardize ground truth protocols [13].

### 9.4. Generalizability and Multi-Site Performance

Generalizability remains a critical barrier to clinical deployment. Thirty-six studies (27.7%) explicitly acknowledge generalizability limitations, particularly related to patient diversity, tissue variability, and acquisition conditions [18]. Skin phototype variability represents one notable example. Melanin absorption significantly affects spectral measurements in the visible and near-infrared range, yet several studies report limited representation of darker skin tones, potentially introducing bias in perfusion and oxygenation estimation [34].

Inter-patient tissue variability further complicates model generalization. Spectral signatures vary with age, comorbidities, hydration, and medication, meaning models trained on homogeneous cohorts may not perform consistently across broader clinical populations. Multi-site acquisition introduces an additional dimension of variability, as differences in hardware, illumination, and calibration procedures alter spectral distributions. The only bi-center clinical HSI study in the review demonstrated reduced cross-center performance compared to within-center evaluation, directly illustrating this generalization gap [17].

### 9.5. Explainability and Clinician Trust

Despite increasing use of deep learning in HSI, explainability remains largely unexplored. Only six studies incorporate interpretability methods such as LIME, Grad-CAM, attention maps, or spectral feature importance visualization [22]. These techniques increase clinicians’ confidence in AI-assisted outputs and help them understand which spectral regions influence classification decisions [57]. The lack of explainability hinders clinical implementation. In high-stakes situations like surgical decision-making, clinicians need to comprehend both the model’s predictions and their underlying assumptions [74]. Systems that cannot convey uncertainty or the rationale behind decisions may be inappropriate for integration into clinical workflows.

Furthermore, emerging regulatory frameworks, including the European Union AI Act and FDA guidance for AI-enabled medical devices, increasingly emphasize transparency and interpretability as requirements for high-risk clinical AI systems [33].

### 9.6. Regulatory and Health Economic Barriers

The majority of HSI research has not addressed regulatory readiness. Only two studies reference regulatory frameworks, including CE-certified HSI systems and national regulatory considerations [98]. No study provides formal regulatory submission evidence, or reimbursement pathway analysis. This highlights a major disconnect between technical development and clinical translation.

The regulatory classification of HSI systems depends on intended use. Systems providing real-time tissue classification for surgical guidance would likely be classified as high-risk medical devices under EU MDR or FDA pathways, requiring large-scale clinical validation. In contrast, perfusion monitoring applications may face lower regulatory thresholds, partially explaining their greater clinical maturity.

Health economic evidence is absent from the reviewed literature. No studies report cost-effectiveness, reimbursement strategies, or workflow integration analysis. Without such evidence, even technically validated systems may face barriers to adoption by healthcare providers and payers. Therefore, successful clinical translation requires addressing economic and regulatory factors in addition to technical development.

## 10. Discussion and Future Directions

The findings of this review reveal a field characterized by rapid algorithmic progress but persistent structural barriers to clinical translation. This section situates these findings within the broader HSI review literature, critically appraises the limitations of both the available evidence and the translational readiness framework proposed here, and derives the translational gaps identified in Section 8 and Section 9.

Prior reviews on medical HSI have addressed the field from technical, domain-specific, or system-level perspectives, but none have provided a cross-domain synthesis framed around clinical evidence quality. Yoon et al. surveyed conventional optical system-based HSI methods [20] but did not organize findings by clinical domain or evaluate diagnostic evidence maturity. Domain-specific reviews have offered greater clinical focus within narrower scopes: Puistenn et al. assessed the level of evidence of machine learning-based HSI in neurosurgery [95], but identified only six eligible publications, limiting the generalizability of their conclusions.

Du et al. reviewed hyperspectral retinal imaging in Alzheimer’s disease and age-related macular degeneration [10], discussing technical advances and clinical bottlenecks without reporting formal inclusion and exclusion criteria. Xiang et al. conducted a systematic review of HSI in gastroenterology and hepatology across 65 studies [112], reporting application-level distributions but not characterizing studies by task formulation or AI/ML methodology.

The present review extends this body of work by synthesizing 130 studies across nine clinical domains, introducing a three-tier translational readiness framework for evidence quality assessment, and systematically extracting validation design, task formulation, and AI/ML methodology, collectively providing a structured, reproducible evidence map not available in prior literature.

The conclusions drawn from this review are constrained by systematic limitations in the underlying literature. The most pervasive is validation-induced performance inflation: patch-based k-fold cross-validation, applied in 51 studies (39.2%), permits intra-patient data leakage that inflates reported accuracy, with studies applying strict inter-patient testing reporting substantially lower performance of approximately 70–80%, compared to the >95% accuracy reported in poorly validated studies. The dominance of ex vivo tissue studies across most domains—including 85% of gastrointestinal and 79% of head and neck oncology studies—further limits the applicability of reported findings to real intraoperative conditions, where motion, blood pooling, and illumination variability substantially alter spectral acquisition. Dataset concentration compounds this issue: only 9 of 130 studies (~7%) provide publicly accessible data, and repeated use of single datasets such as HELICoiD may overestimate algorithmic generalizability. Annotation inconsistency presents an additional constraint, as 78 studies (60%) use histopathology as ground truth at region rather than pixel level, introducing spatial uncertainty into pixel-wise classifiers. Collectively, these limitations mean that cross-study performance comparisons should be interpreted with caution, and the field’s documented performance is likely optimistic relative to real-world deployment outcomes.

The translational readiness framework proposed in this review carries a few inherent constraints. The search strategy captures publications up to early 2026, and studies published beyond this window—particularly those addressing multicenter validation or regulatory evidence—may already partially address the barriers identified here, meaning the tier distribution reflects the field’s maturity at a specific point in time rather than its absolute state. Tier assignment was most challenging at the Tier 2/3 boundary, where studies reporting limited but non-absent external validation are difficult to classify consistently; this is compounded by the fact that approximately 49 studies are described as prospective in their original reporting, yet many represent pilot investigations or preliminary feasibility studies rather than formal clinical trials, making prospective status alone an unreliable indicator of evidence quality. Clinical domain classification presents a further limitation, as boundaries between domains are not always discrete—for example, certain studies characterize tissue type through spatial spectral mapping while others assess perfusion as a surrogate for tissue classification, with both approaches appearing within nominally the same clinical domain. This overlap means that the reported distribution of studies across nine domains should be understood as an organizational framework rather than a precise taxonomy, and the tier distribution and domain breakdowns presented in Section 8 are descriptive rather than inferential.

**Figure 21:**
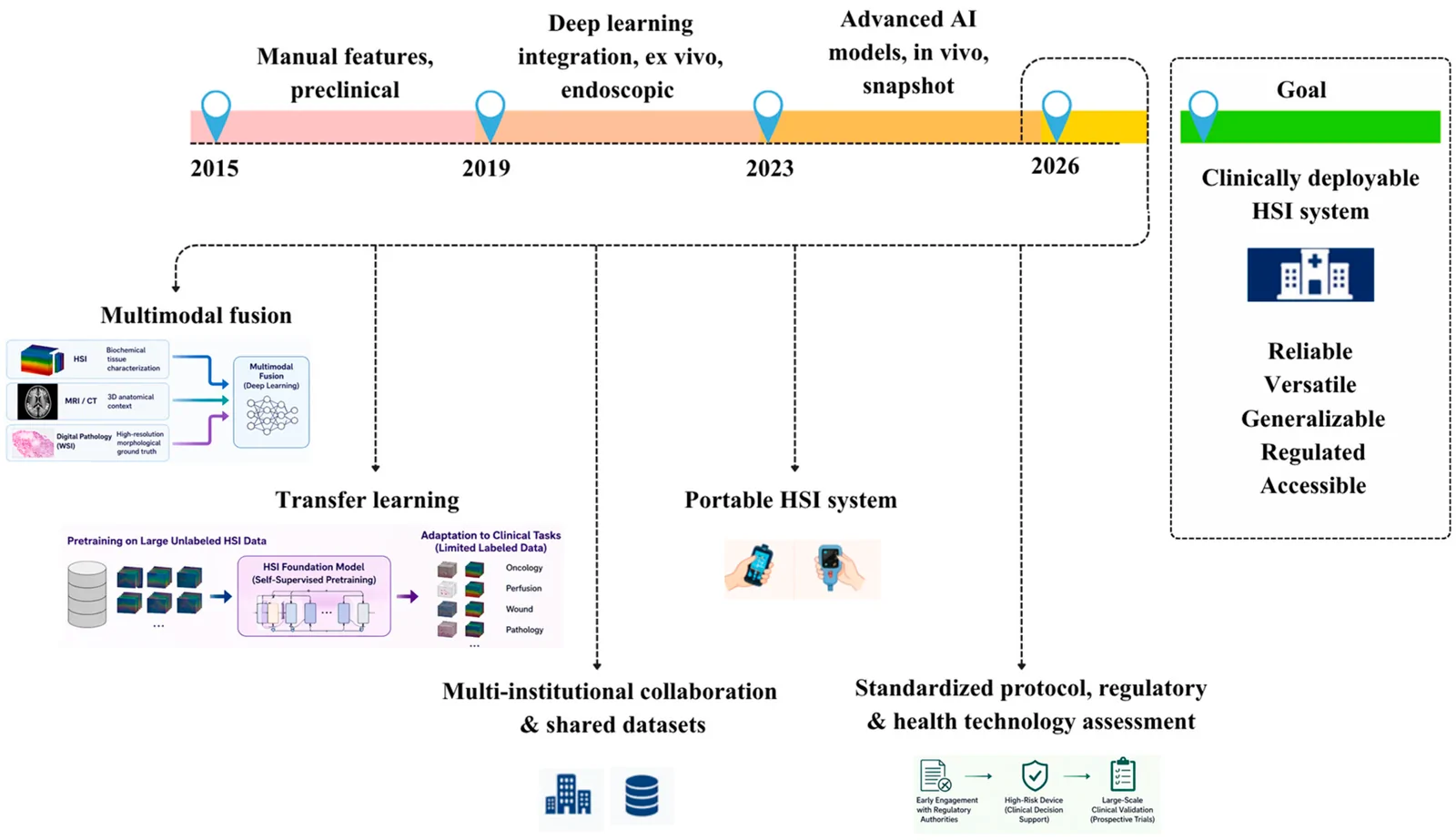
HSI translational roadmap (2015–2026), highlighting key technological milestones and ecosystem requirements for clinical deployment.

These limitations do not change the main findings of this review. The evidence from 130 studies is sufficient to identify clear priorities. Rather than general calls for further research, these priorities target the specific barriers preventing progression from Tier 2 technical validation to Tier 1 clinical deployment. They are organized by translational impact, emphasizing approaches that improve generalizability, dataset scale, clinical integration, and regulatory readiness (Figure 21).

### 10.1. Multimodal Fusion: HSI with Complementary Imaging Modalities

A major approach to improving clinical reliability is multimodal fusion, which combines HSI with complementary anatomical and morphological modalities. Recent studies demonstrate the feasibility of multimodal approaches. Quintana-Quintana et al. introduced a dataset combining HSI, whole-slide imaging, and clinical variables for breast cancer recurrence prediction [41]. Wang et al. proposed HSI-Fusion Net, which integrates spatial and spectral CNN branches for renal carcinoma classification [81]. Du et al. developed a joint diagnostic framework that achieved classification accuracies of 95.46% for gastric cancer and 95.89% for thyroid cancer, outperforming single-modality models [129].

Among potential applications, HSI–MRI co-registration in neurosurgery represents a particularly relevant direction. Integrating intraoperative HSI with MRI-derived anatomical context could provide complementary spatial and biochemical information for surgical guidance and help address the limited anatomical context and penetration depth of HSI identified in Section 7.

### 10.2. Foundation Models and Transfer Learning for Data-Scarce Settings

Limited dataset size and annotation burden remain major barriers to HSI translation. Foundation models and self-supervised learning can address these limitations by learning representations from large volumes of unlabeled spectral data. Recent studies have demonstrated cross-species transfer learning from porcine perfusion data to human organ assessment [92], semi-supervised autoencoders using limited labeled data [85], diffusion-based pre-training for transformer classification with only 5% labeled data [90], and cross-domain adaptation across multiple cancer datasets [74].

These approaches support the development of spectral foundation models trained on heterogeneous HSI data spanning oncology, perfusion, wound imaging, and pathology. Such models could provide more generalizable spectral representations while reducing the need for task-specific labeled data. However, their broader adoption depends on shared multi-institutional datasets, making data harmonization and open benchmarking important prerequisites.

### 10.3. Regulatory Science and Clinical Trial Design for HSI

Despite rapid technical progress, regulatory translation remains largely unexplored. Only isolated studies report CE certification or regulatory considerations, and no work provides formal submission evidence [95]. Therefore, future research must incorporate a regulatory strategy into study design. Planning for future regulatory pathways is essential. Systems providing intraoperative tissue classification would likely fall under high-risk medical device categories, requiring large-scale clinical validation. Early engagement with regulatory frameworks allows studies to simultaneously generate scientific and regulatory-grade evidence [31].

Health economic evaluation represents an additional priority. No studies currently report cost-effectiveness, reimbursement pathways, or workflow impact. Including endpoints such as reoperation reduction, procedure time, and quality-adjusted life years would strengthen clinical adoption. To help with regulatory submissions, standardized clinical trial endpoints for HSI need to be made. These endpoints could include the margin clearance rate, surgeon decision concordance, and workflow efficiency.

### 10.4. Point-of-Care and Portable Hsi Systems

Beyond intraoperative deployment, point-of-care HSI represents a promising pathway for earlier clinical adoption. Portable and handheld systems reduce integration complexity and enable applications in outpatient and bedside settings. Several studies demonstrate feasibility. Lucas et al. explored smartphone-based wound monitoring [130]. Teaw et al. demonstrated a handheld HSI for Raynaud phenomenon assessment [104]. Lin et al. and Dallora et al. applied retinal HSI for dementia biomarker detection in memory clinic environments [15,33].

Spectral reconstruction from RGB imaging is a new area of research. The SAVE method uses the best band combinations to rebuild spectral signatures. This could make it possible to do HSI-like analysis on regular cameras. Chen et al. employed band selection for RGB-based spectral reconstruction in esophageal cancer detection. Further work is needed to validate whether reconstructed spectra retain sufficient biochemical fidelity for clinical decision-making [58].

Collectively, these directions define a structured pathway from algorithmic innovation toward clinically deployable HSI systems.

## 11. Conclusions

This review addressed a question largely absent from the existing literature: not whether hyperspectral imaging can classify tissue accurately, but whether it is prepared for clinical deployment. The answer, derived from a systematic evaluation of 130 studies published between 2015 and 2026, is more nuanced and domain-specific than either the skepticism suggested by limited clinical adoption or the optimistic interpretation of reported accuracy metrics.

The evidence indicates that the field has crossed the technical feasibility threshold in selected domains. In head and neck oncology, the intraoperative utilization of CE-certified endoscopic HSI systems has been evidenced, exhibiting negligible procedural learning curves. In brain and neuro-oncology, validated inter-patient studies, along with integration into certified surgical microscopes, create a reliable intraoperative pathway. In wound and perfusion monitoring, a randomized controlled trial provides the only Level I evidence within the reviewed literature, demonstrating that translation from technical capability to clinical deployment is achievable when evidence generation is aligned with regulatory requirements.

Despite technical progress, HSI lacks the infrastructure required for clinical translation. The tier distribution (~1% near Tier 1, 48.5% Tier 2, 50.8% Tier 3) reflects structural, not technical, limitations. Only five studies are multicenter, eleven include inter-patient validation, and just one randomized controlled trial exists. Explainability is rarely addressed, open datasets are limited, and regulatory engagement is minimal. Overall, the field remains dominated by single-site studies with limited reproducibility and generalizability.

The primary bottleneck is not algorithmic or hardware performance. Deep learning models already achieve clinically relevant inter-patient accuracy, and hardware is increasingly compatible with intraoperative use. Instead, progress is constrained by the absence of standardized validation protocols, device-invariant spectral calibration, multicenter study design, regulatory planning, and health economic evidence. These gaps prevent promising systems from advancing to clinical deployment.

Future progress therefore depends less on new models and more on translational infrastructure. While multimodal fusion, foundation models, and hardware miniaturization remain important, priority should shift toward standardized pipelines, shared calibrated datasets, federated learning, and prospective multicenter trials. Parallel advances in regulatory science and health technology assessment will be essential for achieving clinically approved and deployable HSI systems.

Hyperspectral imaging has established itself as a powerful AI-enabled modality for biochemical tissue characterization. The next phase of development is no longer defined by algorithmic novelty alone but by the construction of the translational ecosystem required for deployment. The evidence synthesized in this review suggests that the technical foundation is already in place; what remains is coordinated progress in validation, standardization, and regulatory alignment. Whether HSI transitions from promising research tool to routine clinical technology will depend less on further performance gains and more on the field’s ability to prioritize reproducibility, interoperability, and clinical integration.

## Figures and Tables

**Table 1:** Inclusion and exclusion criteria.

Criterion	Inclusion	Exclusion
Publication date	January 2015–February 2026	Before January 2015
Study type	Peer-reviewed original research, systematic reviews, and clinical validation studies with quantitative performance metrics	Abstracts only, theses, editorials, non-peer-reviewed papers
Language	English	Non-English publications
Application domain	Medical/clinical HSI in human or large-animal tissue: in vivo, ex vivo, or intraoperative	Remote sensing, agricultural, food quality, phantom-only, or simulation-only studies
Spectral system	HSI with ≥10 contiguous spectral bands, VIS/NIR range (400–1700 nm)	Multispectral (<10 bands), RGB-only, narrowband fluorescence, visible-range-only systems
Outcome reporting	Quantitative diagnostic, classification, or segmentation performance metrics	Qualitative description only: hardware characterization without tissue outcome data

**Table 2:** Four-domain Translational Readiness Appraisal framework.

Domain	Definition	Key Indicators
D1: Hardware OR compatibility	Form factor, acquisition speed, sterility compliance, and illumination requirements compatible with surgical deployment	Acquisition time ≤ 30 s; CE/FDA clearance; endoscope or microscope integration; illumination safety class
D2: Real-time workflow integration	Computational pipeline meets the latency and display requirements of the clinical setting	Inference time ≤ 5 s; minimal OR disruption; clinician proficiency evidence
D3: Cross-site validation	Performance validated across sites, populations, and hardware platforms	Multicenter design, inter-patient validation, device-invariant calibration, population diversity
D4: Regulatory readiness	Evidence base supports the regulatory submission; cost-effectiveness data available	CE/FDA pathway identified; health economic data; reimbursement pathway

**Table 3:** Comprehensive comparison of RGB, multispectral, and hyperspectral imaging modalities in medical applications.

Metric	RGB Imaging	Multispectral Imaging	Hyperspectral Imaging
Conceptual Rendering	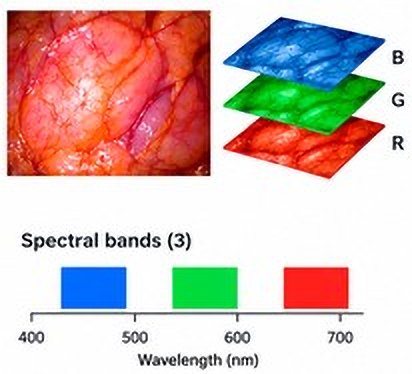	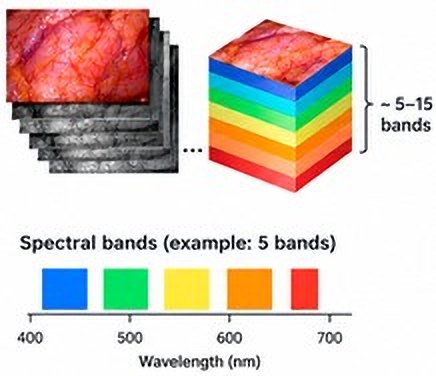	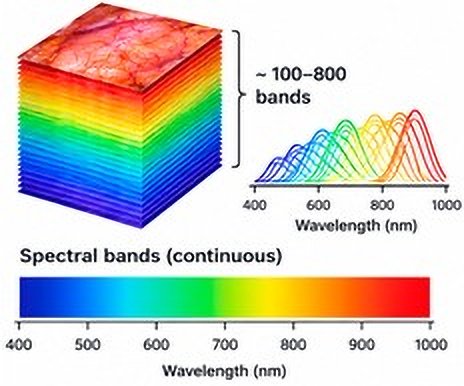
Number of Bands	3 (Red, Green, Blue)	≈4 to 20	Dozens to hundreds (30 to 500+)
Spectral Density	Low: Broad, overlapping channels (≈100 nm width)	Moderate: Discrete, separated bands (≈10–50 nm width)	High: Continuous, tightly packed bands (<1–10 nm width)
Output Data	2D color image (Red, Green, Blue arrays)	Series of discrete 2D spectral images	Deep 3D Data Cube (x, y, λ) with a continuous spectrum per pixel
Clinical Purpose	Morphological context and spatial layout	Targeted biomarker detection (e.g., detecting known fluorophores)	Exploratory biochemical analysis, tumor border detection, subtle tissue change analysis
Limitation	Cannot distinguish spectrally overlapping compounds	Misses information between bands; low spectral resolution	Complex data analysis; high computational cost

**Table 4:** Clinical trade-offs across scanning architectures.

Architecture	Spectral Resolution	Acquisition Speed	Motion Tolerance	Primary Clinical Setting
Pushbroom	High (2–5 nm)	Slow (seconds)	Low	Ex vivo pathology, stabilized OR field
Snapshot	Moderate	Fast (6–50 ms)	High	In vivo, endoscopy, retinal, perfusion
LCTF/AOTF (spectral)	Variable	Slow (~1 min)	Very Low	Controlled lab, ex vivo only

**Table 5:** Summary of data characteristics observed across the reviewed dataset.

Data Characteristics	Key Finding	Share Statistics	Observation
Spectral bands			
Band range	25–826 bands	Median 12 Mode 100	High variability across devices; mode at 100 reflects common pushbroom cameras
Acquisition setting			
Ex vivo-dominant	Ex vivo 60.8%	79/130 studies	Controlled lab conditions limit direct intraoperative translation
In vivo	In vivo 37.7%	49/130 studies	More clinically relevant; smaller share reflects acquisition challenges
Both/Mixed	Mixed 3.1%	4/130 studies	Few studies compare settings directly
Ground truth source			
Histopathology (H&E)	Gold standard	88 studies, 67.7%	Best proxy for tissue classification; still spatially misaligned in many cases
Surgeon/Clinical annotation	Expert label	48 studies, 36.9%	Practical for in vivo; subject to inter-observer variability
Instrument/Reference standard	Device-based	12 studies, 9.2%	Pulse oximetry, radiometry, phantoms—limited biological validity
Not reported/Unclear	Missing	8 studies, 6.2%	Significant reproducibility concerns impede cross-study comparison
Performance metric reported			
Accuracy (Acc%)	Most common metric	89 studies, 68.5%	Often inflated by class imbalance; not sufficient alone
AUC/ROC	2nd most common	53 studies, 40.8%	Preferred for clinical benchmarking; robust to imbalance
Sensitivity/Specificity	Clinically meaningful	31 studies, 23.8%	Inconsistently paired; often reported without confidence intervals
DSC/IoU (segmentation)	Pixel-wise tasks	18 studies, 13.8%	Limited use reflects the low prevalence of spatial segmentation studies
F1-Score	Underused	14 studies, 10.8%	More informative than accuracy metrics, adoption remains low

**Table 6:** Summary of publicly available dataset.

Dataset Name	Domain	Patients (*n*)	Clinical Context	Dataset/Code Link	Related Reference
HELICoiD	Brain/Neuro	13–34	In Vivo	http://hsibraindatabase.iuma.ulpgc.es/ accessed on 4 June 2025.	[22,37]
HeiPorSPECtRaL	Multi-organ	11–20 pigs	Pre-clinical and In vivo	https://www.heiporspectral.org and https://github.com/IMSY accessed on 11 December 2025.	[25,43]
Histology HSI-GB	Brain/Neuro	13	Ex vivo	https://doi.org/10.7937/Z1K6-VD17 accessed on 18 September 2025.	[44,45]
Choledoch (bio-hsi)	GI/Biliary	174	Ex Vivo	http://bio-hsi.ecnu.edu.cn/ accessed on 24 February 2026.	[40]
HistologyHSI-BC-Recurrence (TCIA)	Breast	47	Ex Vivo	https://doi.org/10.7937/6KPY-YT49 and https://github.com/HIRIS-Lab/HistologyHSI-BC-Recurrence accessed on 12 March 2026.	[41]
HMILungDataset	Lung	10	Ex vivo	https://github.com/Intelligent-Imaging-Center/HMILungDataset accessed on 26 February 2026.	[46]
HSPath-Bench	Head and neck	43	Ex Vivo	https://github.com/zhangchenglong1116/HSPath-Bench accessed on 12 March 2026.	[39]
Hyperspectral SRS and Fluorescence data	Rat liver tissue	40–50 images	Ex Vivo	https://doi.org/10.6084/m9.figshare.13497138 accessed on 13 September 2025.	[47]
ColonCancerHSI_Data	Colon adenocarcinoma metastases	14	Ex Vivo	https://data.fulir.irb.hr/islandora/object/irb and https://github.com/ikopriva/ColonCancerHSI accessed on 26 February 2026.	[42]

**Table 10:** Domain-by-domain TRA findings.

Clinical Domain	*n*	Clinical Domain T1/T2/T3	In Vivo Ratio	D1	D2	D3	D4	Overall Tier	Primary Gap	Evidence Needed for Next Tier
Brain/Neuro-oncology	25	1/14/10	60%	~	∼	✕	✕	Tier 2	No multicenter in vivo validation; HELICoiD dataset dependency; no regulatory engagement	Multicenter prospective intraoperative trial; independent dataset validation
Head and neck oncology	19	0/8/11	21%	✓	∼	✕	✕	Tier 2+	Small in vivo cohorts (*N* = 6–12); no multicenter data; CE-certified system used but not replicated	Multi-site prospective trial; *N* ≥ 50 in vivo patients; regulatory submission preparation
GI/Colorectal/Hepatobiliary	47	0/21/25	15%	∼	✕	✕	✕	Tier 2	85% ex vivo; no in vivo endoscopic validation at scale; largest volume, lowest in vivo ratio	In vivo endoscopic validation studies; prospective laparoscopic cohort ≥100 patients
Multi-organ intraoperative	12	0/6/6	83%	✓	∼	∼	✕	Tier 2+	Predominantly porcine; Bannone 2024 [17] is sole bi-center human study; no regulatory pathway identified	Multicenter human intraoperative validation; regulatory classification of multi-organ classifier
Wound/perfusion monitoring	8	0/3/5	100%	✓	✓	∼	∼	Tier 2+	RCT evidence exists but regulatory submission not filed; calibration across skin phototypes absent	Regulatory submission based on existing RCT; skin phototype validation study
Breast oncology	7	0/5/1	14%	∼	✕	✕	✕	Tier 2	Entire evidence base is ex vivo lumpectomy; no intraoperative cavity imaging study exists	First in vivo intraoperative lumpectomy cavity HSI study; snapshot hardware development
Gastroenterology/Endoscopy	7	0/2/2	29%	∼	∼	✕	✕	Tier 2	No video-rate endoscopic HSI system commercially available; SAVE approach invalidated clinically	Integrated snapshot endoscopic HSI or clinically validated SAVE reconstruction; prospective endoscopy trial
Dermatology	6	1/1/4	33%	✓	✓	✕	✕	Tier 2	Small cohorts (*N* = 11–34); limited skin phototype diversity; no multi-site study	Multi-site prospective skin lesion study; diverse phototype cohort; regulatory pathway identified
Emerging/Other	23+	0/1/22	varies	✕	✕	✕	✕	Tier 3	All applications proof-of-concept; no domain has reached inter-patient validation at scale	Retinal neuro degeneration and gynecologic oncology are strongest candidates for coordinated multi-site investment

*n* = number of included studies per domain; in vivo ratio indicates studies using in vivo human or large-animal data; D1–D4 denote translational readiness domains: D1—hardware/OR compatibility; D2—real-time workflow integration; D3—cross-site validation; D4—regulatory and cost-effectiveness readiness; T1–T3 show counts by tier, and Tier 2+ denotes Tier 2 with ≥1 domain achieved. Symbols: ✓ met, ∼ partial/single-study, ✕ not met. See Section 2.5 for details.

## Data Availability

No new data were created or analyzed in this study. Data sharing is not applicable to this article.
